# Common and Rare Variants in *TMEM175* Gene Concur to the Pathogenesis of Parkinson’s Disease in Italian Patients

**DOI:** 10.1007/s12035-022-03203-9

**Published:** 2023-01-07

**Authors:** Nicole Piera Palomba, Giorgio Fortunato, Giuseppe Pepe, Nicola Modugno, Sara Pietracupa, Immacolata Damiano, Giada Mascio, Federica Carrillo, Luca Giovanni Di Giovannantonio, Laura Ianiro, Katiuscia Martinello, Viola Volpato, Vincenzo Desiato, Riccardo Acri, Marianna Storto, Ferdinando Nicoletti, Caleb Webber, Antonio Simeone, Sergio Fucile, Vittorio Maglione, Teresa Esposito

**Affiliations:** 1 IRCCS INM Neuromed, Pozzilli, IS Italy; 2 Institute of Genetics and Biophysics, Adriano Buzzati-Traverso”, National Research Council, Naples, Italy; 3 Department of Environmental, Biological and Pharmaceutical Sciences and Technologies (DiSTABiF), University of Campania Luigi Vanvitelli, Caserta, Italy; 4 Dementia Research Institute, Cardiff University, Cardiff, UK; 5 Department of Physiology and Pharmacology, Sapienza University of Rome, Rome, Italy; 6 Oxford Parkinson’s Disease Centre, Department of Physiology, Anatomy, Genetics, University of Oxford, Oxford, UK

**Keywords:** Parkinson’s disease, *TMEM175*, Association study, Mutation analysis, Electrophysiology analysis, Autophagy-lysosomal pathway

## Abstract

Parkinson’s disease (PD) represents the most common neurodegenerative movement disorder. We recently identified 16 novel genes associated with PD. In this study, we focused the attention on the common and rare variants identified in the lysosomal K^+^ channel TMEM175. The study includes a detailed clinical and genetic analysis of 400 cases and 300 controls. Molecular studies were performed on patient-derived fibroblasts. The functional properties of the mutant channels were assessed by patch-clamp technique and co-immunoprecipitation. We have found that *TMEM175* was highly expressed in dopaminergic neurons of the substantia nigra pars compacta and in microglia of the cerebral cortex of the human brain. Four common variants were associated with PD, including two novel variants rs2290402 (c.-10C > T) and rs80114247 (c.T1022C, p.M341T), located in the Kozak consensus sequence and TM3II domain, respectively. We also disclosed 13 novel highly penetrant detrimental mutations in the *TMEM175* gene associated with PD. At least nine of these mutations (p.R35C, p. R183X, p.A270T, p.P308L, p.S348L, p. L405V, p.R414W, p.P427fs, p.R481W) may be sufficient to cause the disease, and the presence of mutations of other genes correlated with an earlier disease onset. In vitro functional analysis of the ion channel encoded by the mutated *TMEM175* gene revealed a loss of the K^+^ conductance and a reduced channel affinity for Akt. Moreover, we observed an impaired autophagic/lysosomal proteolytic flux and an increase expression of unfolded protein response markers in patient-derived fibroblasts. These data suggest that mutations in *TMEM175* gene may contribute to the pathophysiology of PD.

**Supplementary Information:**

The online version contains supplementary material available at 10.1007/s12035-022-03203-9.

## Introduction

Parkinson’s disease (PD) is a common neurodegenerative disorder characterized by a wide spectrum of motor symptoms (bradykinesia, muscular rigidity, resting tremor, and postural instability) which are caused by the progressive loss of dopaminergic (DA) neurons in the substantia nigra pars compacta (SNpc) [1, 2]. The pathological hallmark of PD is the presence of Lewy bodies containing alpha-synuclein aggregates [3] in some surviving SNpc neurons [4].

In the last years, genome-wide association studies (GWAS) and next-generation sequencing (NGS) analysis of large cohorts of familiar and sporadic cases identified a number of genes/variants associated with PD; however, for the majority of them, the causal role in the pathogenesis of PD has not yet been identified [5–7].

In a recent study, we identified 16 novel candidate genes for PD in Italian families. These genes are expressed in the mesencephalon and are involved in pathways, controlling mitochondrial metabolism and oxidative stress, vesicular trafficking, microtubule dynamics, and autophagy, all pathways potentially deregulated in PD [8]. We also demonstrated that polygenic inheritance of rare variants in PD Mendelian genes might predict the disease occurrence in both familial and sporadic cases highlighting the importance of rare variants also in the genetics of sporadic PD [8].

*TMEM175*, one of the novel genes identified in our cohort of PD families, is located in a highly significant PD GWAS peak at chromosome 4 [9, 10]. Recently, two common coding variants, rs34884217 (c.A194C, p.Q65P) and rs34311866 (c.T1178C, p.M393T), have been associated with PD risk in several populations [11–14]. This gene encodes for an endolysosomal K^+^ channel that is involved in K^+^ conductance, pH stability, and α-synuclein clearance [15–18]. Deficiency in TMEM175 leads to the loss of dopaminergic neurons and impairment in motor function in mice [19].

TMEM175 is a protein of great interest for PD etiopathogenesis because it might be potentially modulated by pharmacological agents. However, a detailed genetic analysis is still missing.

In this study, we performed a comprehensive molecular and genetic analysis in PD Italian patients. We identified novel common and rare variants associated with PD and examined their potential role in the pathophysiology of the disease.

## Patients and Methods

### Study Participants

Four hundred PD patients (243 males and 157 females; 178 familiar and 222 sporadic cases) were recruited at the Istituto di Ricovero e Cura a Carattere Scientifico (IRCCS, Scientific Institute for Research, Hospitalization and Healthcare) Mediterranean Neurological Institute (MNI) Neuromed in Pozzilli, here called MNI cohort. The project was approved by the ethical committees of IRCCS Neuromed, and written informed consent was signed by all participants. All subjects were of European ancestry and were evaluated by qualified neurologist of the Parkinson Centre from June 2015 to December 2017, with a thorough protocol comprising neurological examination and evaluation of non-motor domains. Information about family history, demographic characteristics, anamnesis, and pharmacological therapy was also collected [8, 20, 21].

The Movement Disorder Society revised version of the Unified Parkinson’s Disease Rating Scale Part III (18 items, maximum score 72; hereafter called UPDRS) [22] was used for the assessment of clinical motor symptoms. These included language, facial expressions, tremor, rigidity, agility in movements, stability, gait, and bradykinesia. Cognitive abilities were tested through an Italian validated version of the Montreal Cognitive Assessment (MoCA) [23]. The assessed cognitive domains include short-term memory (5 points); visuospatial abilities via clock drawing (3 points) and a cube copy task (1 point); executive functioning via an adaptation of Trail Making Test Part B (1 point), phonemic fluency (1 point), and verbal abstraction (2 points); attention, concentration, and working memory via target detection (1 point), serial subtraction (2 points), digits forward and backward (1 point each); language via confrontation naming with low-familiarity animals (3 points), and repetition of complex sentences (2 points); and orientation to time and place (6 points). The total score was given by the sum of these domains and then divided by the maximum score that could be obtained (30 points). Where one or more domains could not be tested (e.g., visuospatial tasks, due to unavailability of optical devices), the corresponding score was subtracted from the total score obtainable.

Non-motor symptoms were assessed through an Italian-validated version of Non-motor Symptoms Scale (NMMS) for Parkinson’s disease [24]. This scale tests 9 items, including cardiovascular domain, sleep/fatigue, mood/cognition, perceptual problems/hallucinations, attention/memory, gastrointestinal, urinary, sexual function, and ability to taste or smell. For each item, both severity and frequency of symptoms are measured, so that the scale accounts for both aspects. Here, the sleep domain was slightly modified by adding a further question on the occurrence of vivid dreams. This question was treated like all the others, i.e., the severity of impairment was scored from 0 (no symptoms) to 3 (severe impairment), and the frequency of impairment was scored from 0 (less than once a week) to 4 (daily impairment); then the total score of the sub-item was computed as the product of severity by frequency and added to the scores of the other sub-items.

A subset of participants carrying deleterious variants in *TMEM175* gene underwent a standardized magnetic resonance imaging (MRI) protocol with a 3 T SIGNA HD platform (United States General Electric Medical Group, GE Healthcare) including the following sequences: high-resolution 3-dimensional T1-weighted (T1-3D) magnetization-prepared rapid acquisition with gradient echo sequence (repetition time [TR] = 1900 ms, echo time [TE] = 2.93 ms, flip angle = 9, field of view [FOV] = 260 mm, matrix = 256 × 256, 176 contiguous sagittal 1-mm thick slices) and dual turbo spin-echo proton density and T2-weighted images (TR = 3320 ms, TE = 10/103 ms, FOV = 220 mm, matrix = 384 × 384, 25 axial 4-mm thick slices, 30% gap).

Whole-exome sequencing (WES) data were available for 112 PD cases. WES was performed by using the SureSelect All Exome kit v6 (Agilent® Technologies, Santa Clara, CA, USA) for DNA fragmentation and capture. Exomes were barcoded and sequenced at Helmholtz Zentrum, München, Germany, using the Illumina® HiSeq2000 platform.

Next-generation sequencing-targeted resequencing (NGS-TR) was performed on 288 PD cases. Probes specific for 100 genes, as described previously [8], were designed with Nimble Design software (Roche Diagnostics, Mannheim Germany). Targeting regions were enriched using the SeqCap kit based on DNA fragmentation (Kapa Hyper plus kit) and capture (Roche Diagnostics, Mannheim Germany). Targeted regions were barcoded and sequenced on MiSeq platform (Illumina, San Diego, CA, USA).

Italian control cohort included whole genome data of 107 samples of the Tuscan Italians (TSI) population of the 1000 Genome Project (phase 3 release) and NGS-TR data of 192 healthy subjects from Neuromed biobank [8].

### Human Dermal Fibroblast (hDF) Cell Lines

The hDF cells were generated in our laboratory. Skin samples of PD patients and unrelated healthy individuals were obtained by skin punch biopsy mostly at the inner side of the upper arm. Tissue pieces were enzymatically digested for 5 h at 37 °C by using Collagenase/Dispase kit (Roche Diagnostics, Mannheim Germany), followed by mechanically disaggregation with knife. Cells were grown in Dulbecco’s modified Eagle’s medium (DMEM) supplemented with 4.5 g/L D-glucose, 0.11 g/L sodium pyruvate, 20% FBS, 1% NEAA, 1% L-glutamine, and 1% Pen-Strep at 37 °C in a humidified CO_2_ 5% air for about 4–5 weeks. hDF cell lines were maintained and were used in all the experiments in sub-confluent monolayers. Starvation was performed for 1 h in HBSS. In bafilomycin treatment, cells were starved for 2 h in HBSS with/without 100 mM bafilomycin.

### Plasmid Preparation, Cell Culture, and Transfection

The human wild-type (wt) *TMEM175* construct was purchased at Source Bioscience and was used as template for PCR reaction to obtain the full-length cDNA fragment that was introduced into the pCagMCSEGFPiresNEO expression vector to generate a GFP-tagged TMEM175 construct. The mutagenesis of the wt cDNA to obtain mutant forms (c.C103T, p.R35C; c.G808A, p.A270T; c.C923T, p.P308L; c.T1022C, p.M341T; c.C1213G, p.L405V) was carried out by using the Quick Change II XL site-directed mutagenesis technique according to the manufacturer’s instructions (Agilent Technologies, Santa Clara, CA, USA). The coding region of wt and mutant plasmids was sequenced to confirm the mutagenesis and to exclude undesired mutations.

The mouse dopaminergic neuronal cell line, MN9D, was cultured in high-glucose Dulbecco’s modified Eagle’s medium (DMEM) supplemented with 10% FBS (Thermo Fisher Scientific, Waltham, MA, USA) and 2% L-glutamine in a humidified atmosphere of 5% CO_2_ at 37 °C.

Ten micrograms of the constructs were linearized and electroporated in MN9D cells. Stable transfected clones were grown and selected by puromycin. The mutant and wt clones were analyzed by WB and immunoprecipitation (IP) assay.

Human embryonic kidney 293 (HEK293) cells and HeLa cells were cultured in Dulbecco’s modified Eagle’s medium (DMEM) with 4.5 g/L D-glucose, 0.11 g/L sodium pyruvate, 10% FBS (Thermo Fisher Scientific, Waltham, MA, USA), 1% L-glutamine, and 1% Pen-Strep at 37 °C in a humidified atmosphere of 5% CO_2_. Ten micrograms of the constructs were electroporated in HEK293 cells for transient transfection.

### Patch-Clamp Experiments

HEK293 cells transfected with the GFP-tagged wt or mutant proteins were plated (80,000 cells) on 35-mm tissue culture dishes 24/48 h before the experimental day. Currents were recorded with a Multiclamp 700B amplifier and Clampex 10.5 software (Molecular Devices, San Jose, CA, USA) in the perforated patch-clamp configuration to reduce the perturbation of the intracellular milieu, by using escin (50 μM). Patch-clamp recordings were obtained using borosilicate glass electrodes (4–6 MΩ) filled with intracellular solution contained (in mM) 140 CsMetSO_3_, 5 BAPTA-AM, 2 MgCl_2_, 2 MgATP, 10 HEPES buffered with CsOH, and pH = 7.4. During recordings, cells were continuously perfused using normal external solutions (NES), containing (in mM) 140 Na-gluconate (Na-Glu), 2.8 KCl, 2 MgCl_2_, 2 CaCl_2_, 10 glucose, 10 HEPES buffered with NaOH, and pH 7.4. Cells were held at − 70 mV, and currents were elicited with voltage ramps (from − 100 to + 100 mV, 200 ms) every 2 s. The current density at + 30 mV was determined as the current amplitude/cell capacitance (pA/pF) [15]. Cell capacitance was continuously monitored.

Data analysis was performed using SigmaPlot 14.0 (Systat). All data are expressed as means ± standard error mean and analyzed using Student’s t-test. Significance for all tests was set at *P* < 0.05.

### Western Blot and Immunoprecipitation (IP) Assay

hDF cells were collected and washed with cold PBS (2.7 mM KCl, 1.2 mM KH_2_PO_4_, 8.1 mM Na_2_HPO^4^, 138 mM NaCl [pH 7.4]). Total proteins were solubilized in lysis buffer (20 mM Tris–HCl [pH 7.4], 1 mM ethylenediaminetetraacetic acid (EDTA), 0.4 mM NaF, 0.04 mM Na_3_VO, 1% NP40, protease inhibitors) and sonicated in ice. Extraction of separate cytoplasmic and nuclear protein fractions was performed with NE-PER Nuclear and Cytoplasmic Extraction Kit (Thermo Fisher Scientific, Waltham, MA, USA). Proteins were separated by SDS-PAGE and transferred on PVDF membrane (Millipore, Bedford, Massachusetts, USA). The level of expression of TMEM175, LC3I/II, p62, LAMP1, TFEB, GAPDH, and PARP1 was determined by immunoblot analysis using anti-LAMP1 (ab25630, Abcam, Cambridge, UK, 1:1000), anti-LC3B (2775, Cell Signaling, Danvers, Massachusetts, USA, 1:1000/), anti-TMEM175 (19,925–1-AP, Proteintech, Manchester, UK, 1:1000) and anti-p62/SQSTM1 (P0067, Sigma-Aldrich, St. Louis, MO, USA, 1:15,000), anti-TFEB (A303-673A, Bethyl Laboratories (Waltham, MA, USA), 1:1000), anti-PARP1 (66,520-1Ig, Proteintech, Manchester, UK, 1:20,000), and anti-GAPDH (sc-32233, Santa Cruz Biotechnology (Dallas, Texas USA), 1:1000). Cytoplasmic proteins were normalized to GAPDH, whereas nuclear proteins were normalized to PARP1.

Immunoprecipitation (1 mg total lysate) was performed using the anti-Akt antibodies (Immunological Sciences, Cat. N. MAB-94320) complexed to protein G-Sepharose (Invitrogen, Cat. N. 101,243). The immunoprecipitated proteins were resolved on 10% SDS–PAGE, transferred for 2 h at room temperature on PVDF membrane, and detected by immunoblotting with GFP (1:1000) (Synaptic System, Cat. N. 132 002) and Akt (1:1000). HRP-conjugated secondary antibodies (Immunological Sciences, Cat. N. IS20402) were used at 1:5000 dilution. Protein bands were detected by ECL and visualized by Quantity One software (Bio-Rad Laboratories).

The mean standard deviations of 3 independent experiments were analyzed as multiple datasets with ANOVA test for multiple comparisons or with Student’s t-test. Data were plotted as histogram representation. A value of *p* < 0.05 was considered statistically significant.

### Expression Studies

Data of the human brain (cortex and substantia nigra) single-cell expression profile were from Webber laboratory [25]. Total RNA from human adult tissues was from Stratagene (La Jolla, CA, USA); total RNA from hDF was isolated from 10^6^ cells; midbrain, cortex, striatum, and hippocampus RNAs were extracted from three adult mice (P45). RNA was isolated by using EuroGold Trifast kit (Euroclone, IT) protocol. Five micrograms of total RNA was digested with TURBO™ DNase (RNase free) kit (Thermo Fisher Scientific, Waltham, MA, USA) to eliminate genomic DNA contamination. Two micrograms of DNA-free RNA were reverse transcribed with the Superscript III-First strand kit (Thermo Fisher Scientific, Waltham, MA, USA). Quantitative PCR (qPCR) reactions were performed in triplicate, using gene specific primers (Table S1) and ITaq Universal Sybr Green Supermix (Bio-Rad, Hercules, CA, USA) following the manufacturer’s directions. Results were normalized to the expression of the glyceraldehydes-3-phosphate dehydrogenase (GAPDH) gene. Standard deviation was calculated by using data of three different experiments.

### Immunofluorescence Staining of hDF Cell Lines

hDF cells (20,000 cells in 24-well plates on coverslip) were grown in complete medium for 24 h. Starvation was performed for 1 h in Hanks’ Balanced Salt solution (HBSS) before fixing with paraformaldehyde (PFA) 4% and staining with anti-LAMP1, anti-LAMP2, anti-p62, anti-LC3B, anti-TMEM175, anti-GC, and anti-GM antibodies. Briefly, incubation with blocking buffer (1 × PBS/0.1% triton/5% serum) was performed for 15 min in a humidified chamber. Primary and then secondary antibody incubations (1 × PBS/0.1% triton/antibody) were performed at 4 °C overnight and at RT for 1 h, respectively. Anti-LAMP1 (ab25630, Abcam, Cambridge, UK, 1:20), anti-LAMP2 (ab25631, Abcam, Cambridge, UK, 1:100), anti-LC3B (2775, Cell Signaling, Danvers, MA, USA, 1:200), anti-TMEM175 (19,925–1-AP, Proteintech, Manchester, UK, 1:100) and anti-p62/SQSTM1 (P0067, Sigma-Aldrich, St. Louis, MO, USA, 1:500), anti-GlcCer (RAS0010, Glycobiotech, Kuekels, Germany, 1:50) primary antibodies and anti-mouse IgG-Cy3 (C2181, Sigma-Aldrich, St. Louis, MO, USA, 1:200), anti-rabbit IgG-Cy3 (C2306, Sigma-Aldrich, St. Louis, MO, USA, 1:200), Donkey anti-Mouse IgG (H + L), Alexa Fluor™ 488 (A-21202, Thermo Fisher Scientific CA USA, 1:400), and Donkey anti-Rabbit IgG (H + L) Alexa Fluor™ 488 (A-21206, Thermo Fisher Scientific CA USA, 1:400) secondary antibodies were used. GM1 was detected through 2-h incubation with Cholera toxin subunit B (CT-B) conjugated with Alexa fluor 488 (c34775, Molecular Probes, Eugene, OR, USA, 1:50). Hoechst H3570 (Thermo Fisher Scientific, CA, USA, 1:1000) was used to visualize the nuclei. Slides were visualized with Nikon Confocal Microscope A1R and with Nikon Eclipse Ni-E Microscope.

### Immunohistochemistry Assays

Brain tissues were obtained from control animals of 3 months of age, previously authorized in the project 545/2019-PR. Immunohistochemistry was performed on paraformaldehyde-fixed, wax-included brains as described previously [26]. In brief, slides were xylene deparaffinized and rehydrated through a descending series of alcohol to water before being boiled in citrate buffer (pH 6.0) for antigen retrieval. Sections were then incubated with a blocking solution containing 0.5% milk, 10% FBS, and 1% BSA and hybridized with the primary and Alexa Fluor secondary antibodies (Molecular probes, Eugene, Oregon, USA). Primary antibodies were rabbit anti-Tmem175 (1:200, bs-18922R Bioss Inc, Boston, MA, USA), mouse anti-GFAP (1:200, MAB3402 Chemicon, Temecula, CA, USA), mouse anti-NeuN (1:100, MAB377 Chemicon, Temecula, CA, USA), and Chicken anti-Iba1 (1:2000, 234,009 Synaptic Systems, Goettingen, Germany). The immunohistochemistry experiment to visualize Tmem175, Iba1, and NeuN was performed with three compatible antibodies; fluorescence was excited at 488 nm (Tmem175, green), 555 nm (NeuN, red), and 647 nm (Iba1, red) for secondary antibodies. All immunostaining images were captured with a Nikon eclipse NI microscope.

### Statistical Analysis

Categorical variables were compared with χ2-test or Fisher’s exact test, as appropriate. Genotype and allele frequencies were computed, and their distribution in cases and controls was analyzed by χ2-test with 2 DF and 1 DF. Concordance to the frequency predicted by the Hardy–Weinberg equilibrium (HWE) was assessed by χ2-test with 1 DF.

SPSS statistical software (SPSS Inc., Chicago, IL, USA, version 12.0) was used for most of the statistical analysis. Tests for deviation from Hardy–Weinberg equilibrium and tests for association were performed with DeFinetti (http://ihg.gsf.de/cgi-bin/hw/hwa1.pl). The difference between the observed means was calculated with MedCalc (https://www.medcalc.org/calc/comparison_of_means.php). The power of our case–control sample was calculated by the QUANTO statistical software. This study had the ability to detect, assuming 80% power and a two-sided 0.05 significance level with a minor allele frequency of 20% and an odds ratio > 1.51 (https://www.stat.ubc.ca/~rollin/stats/ssize/caco.html). All calculations were considered significant for *P* < 0.05. Bonferroni correction for the number of SNP was tested (*α* = 0.05/7 = 0.007).

In the endophenotype analysis, we used UPDRS score < 30 (corresponding to a mild phenotype: 1–2 of Hoehn and Yahr (HY) scale) and ≥ 30 (corresponding to a severe phenotype: 3–5 HY scale) [27], while in non-motor symptoms, we used as cutoff ≤ 54 and > 54 (9 items × 2 (mild impairment) × 3 (weekly impairment)). Statistical significance was set to *α* = 0.01, correcting for five phenotypes tested.

## Results

### Expression Profile of *TMEM175* Gene in Human and Mouse Tissues

**Fig. 1:**
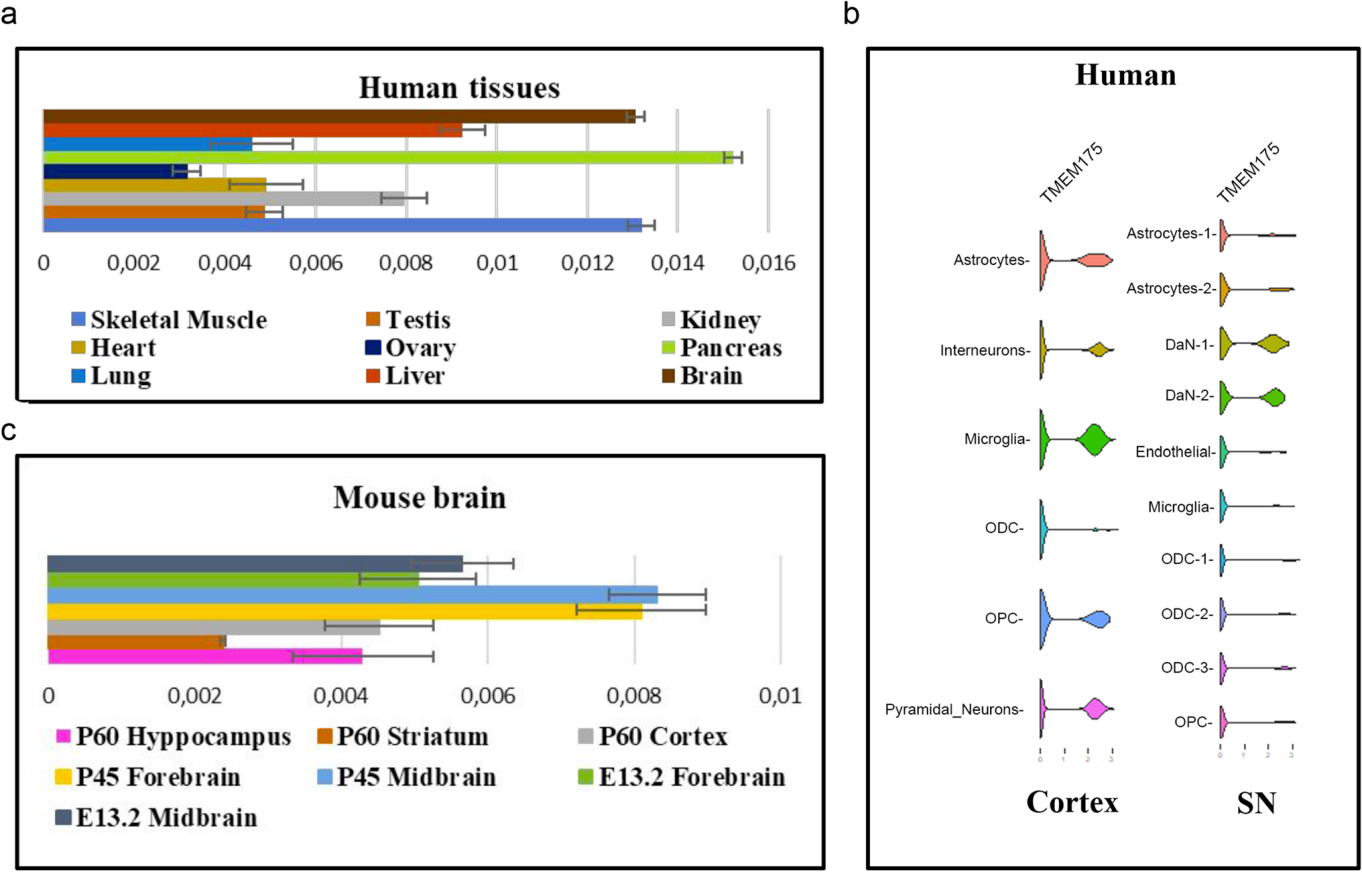
Expression analysis of *TMEM175* in human and mouse tissues. **a**,** b** qPCR experiments showed that *TMEM175* mRNA is highly expressed in the human brain (brown bar) and mouse midbrain and forebrain (blue and yellow bar) at P45. Results were normalized by *GAPDH*. Data are expressed as absolute values with SD calculated from three biological replicates. **c** Single-cell RNA sequencing analysis of human substantia nigra (SN) and cerebral cortex showed the highest expression of *TMEM175* mRNA in dopaminergic neurons of SN and in microglia of the cerebral cortex. Abbreviations: SN, substantia nigra; ODC, oligodendrocytes; OPC, oligodendrocyte progenitor cell

**Fig. 2:**
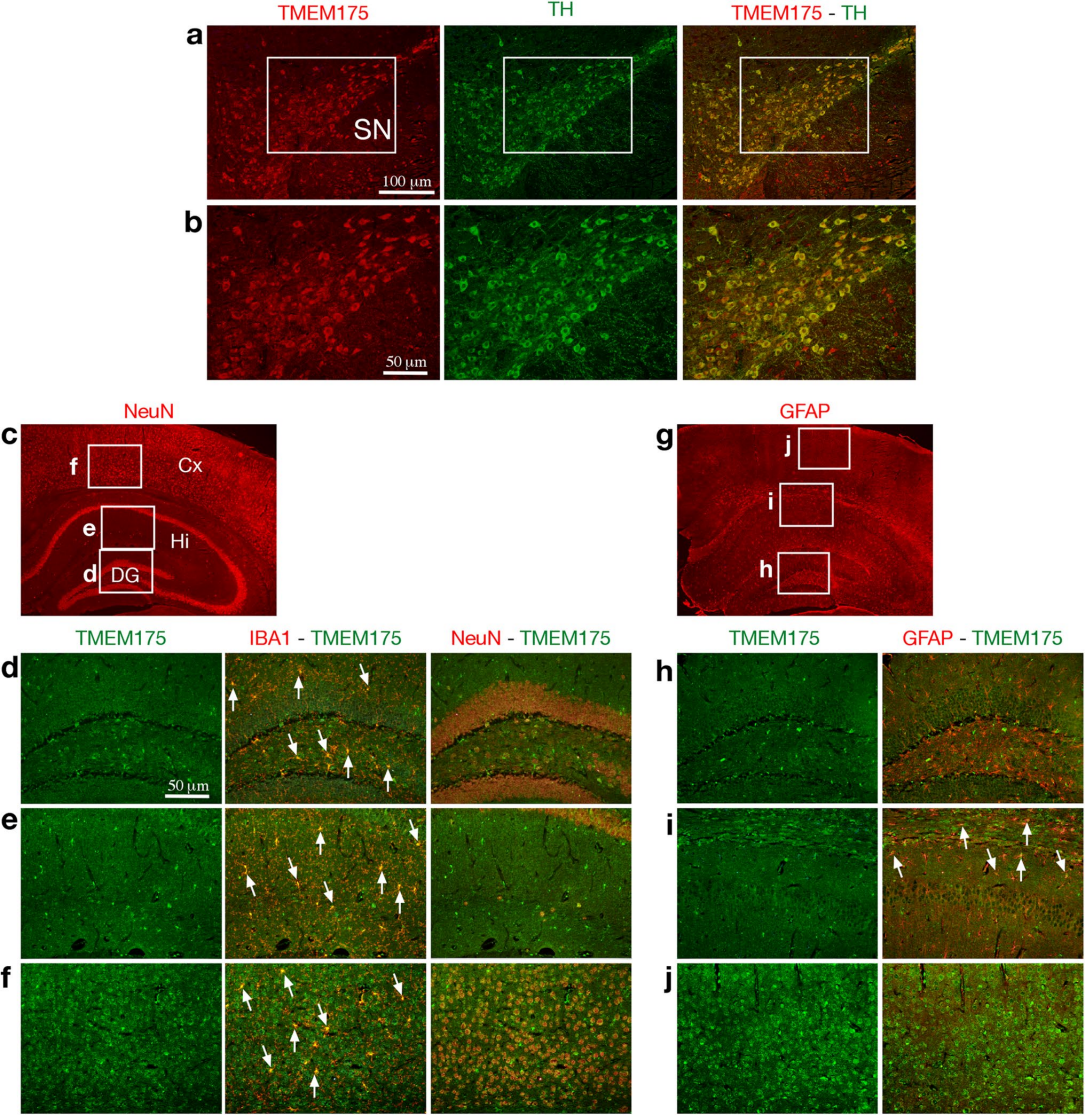
TMEM175 expression in the substantia nigra and dorsal telencephalon of the mouse adult brain. **a–j** Immunohistochemistry assays performed on mouse adult brain sections with Tmem175 and TH (**a**, **b**); NeuN (**c**); Tmem175, IBA1, and NeuN (**d–f**); GFAP (**g**); and Tmem175 and GFAP (**h–j**) shows that in the substantia nigra Tmem175 protein is co-expressed with TH in all dopaminergic neurons (**a**, **b**); in the dorsal telencephalon, Tmem175 exhibits high expression level in IBA1^+^ microglia cells (arrows in **d–f**) and a lower expression level in virtually all NeuN^+^ cortical neurons (**f**) and in a small fraction of GFAP^+^ putative astrocytes prevalently distributed in a territory including the oriens layer of the hippocampus, the alveus, and the white matter of corpus callosum (arrows in **i**). The area demarcated in (**a**) corresponds to sections shown in (**b**), and those demarcated in (**c**) and (**g**) correspond to sections shown in (**d–f**) and (**h–j**), respectively. Abbreviations: SN, substantia nigra; Cx, cortex; Hi, hippocampus; DG, dentate gyrus. Scale bars are indicated

Quantitative PCR analysis showed that the transcript of *TMEM175* gene was ubiquitously expressed in both adult human and mouse brain tissue (Fig. 1a, c). A higher expression was observed in the brain, pancreas, and skeletal muscle in human (Fig. 1a) and in midbrain and forebrain in the mouse brain (Fig. 1c). Analysis of human single-cell RNA sequencing data [25] showed the highest *TMEM175* expression in dopaminergic neurons (DaN_1 and DaN_2) of the SNpc and in microglia of the cerebral cortex (Fig. 1b). In mouse brain, the highest expression of Tmem175 protein was observed in all DA neurons (TH + cells) of the SNpc (Fig. 2a, b) and in microglia (Iba1 + cells) of the dentate gyrus, hippocampus, and cortex (Fig. 2d, e, f). A lower expression was also detected in cortical neurons (NeuN + cells) (Fig. 2f) and in astrocytes (GFAP + cells) in the hippocampus (Fig. 2i). Overall, these data suggest a role for *TMEM175* in the etiopathogenesis of PD and neuroinflammation.

### Comprehensive Molecular Analysis of *TMEM175* Gene in Italian Late-Onset PD (LOPD) Patients

The *TMEM175* gene is located on chromosome 4 in the TMEM175/GAK/DGKQ GWAS locus associated with PD [9]. Recently, the TMEM175 p.M393T common variant was described as a risk factor for PD in several populations [11, 14], and rare deleterious variants were associated with PD in Italian families [8].

To assess the genetic contribution of *TMEM175* gene in the Italian population, we performed a retrospective observational monocentric study of 400 PD patients (178 familiar and 222 sporadic cases; mean age at diagnosis 58.26 years (standard deviation (SD) 9.74)) [8, 20] and 300 unrelated healthy subjects (mean age 77 years; SD 5.4). We found 66 variants including 34 exonic (12 synonymous, 20 non-synonymous, 1 frameshift deletion, and 1 stopgain) variants, 9 variants in untranslated (3′ and 5′) regions, and 23 variants in intronic regions adjacent to the exon/intron boundaries. The complete list of identified variants is reported in Table 1.

**Table 1:** *TMEM175* variants annotated in Italian cohort of patients and controls

Chr	POS	dbSNP	REF	ALT	Position	Gene	Type of variant	Transcript	Nt change	AA change	MAF ItalianPD	MAF Italian CNT	MAF max in public datasets
chr4	926,218		G	A	UTR5	TMEM175		NM_032326	c.-15310G > A		0.0017	0	0
chr4	926,270	rs750230582	C	G	UTR5	TMEM175		NM_032326	c.-15258C > G		0	0.005	0
chr4	939,883	rs7687129	T	C	Intronic	TMEM175					0.7	0.7	0.7
chr4	939,898		G	A	Intronic	TMEM175					0	0.005	0
chr4	939,908	rs145572387	G	C	Intronic	TMEM175					0.007	0	0.003
chr4	941,518	rs2290402	C	T	UTR5	TMEM175		NM_032326	c.-10C > T		0.27	0.2	0.16
chr4	941,551	rs34555481	G	A	Exonic	TMEM175	SV	NM_032326	c.G24A	p.E8E	0.005	0	0.0177
chr4	941,568	rs148512239	C	T	Exonic	TMEM175	NSV	NM_032326	c.C41T	P14L	0.0024	0.005	0.0029
chr4	941,587	rs142678768	C	A	Exonic	TMEM175	SV	NM_032326	c.C60A	p.G20G	0.0017	0.0024	0.0013
chr4	941,590		G	T	Exonic	TMEM175	NSV	NM_032326	c.G63T	p.R21S	0	0.005	0
chr4	941,630	rs542936413	C	T	Exonic	TMEM175	NSV	NM_032326	c.C103T	p.R35C	0.0034	0	0.008
chr4	941,650	rs34645349	C	T	Exonic	TMEM175	SV	NM_032326	c.C123T	p.D41D	0.004	0.005	0.0012
chr4	941,718	rs371616631	G	A	Intronic	TMEM175					0.01	0	0.0001
chr4	942,006	rs2290403	G	A	UTR5	TMEM175		NM_001297423	c.-2257G > A		0.3	0.33	0.36
chr4	942,092	rs543933914	G	T	UTR5	TMEM175		NM_001297423	c.-2171G > T		0.0017	0	0.001
chr4	942,132	rs754596846	C	A	UTR5	TMEM175		NM_001297423	c.-2131C > A		0.0017	0	0
chr4	942,190	rs10516157	C	G	UTR5	TMEM175		NM_001297423	c.-2073C > G		0	0.01	0.06
chr4	942,282		C	T	UTR5	TMEM175		NM_001297423	c.-1981C > T		0.0017	0	0
chr4	944,132	rs755406240	CT	C	Intronic	TMEM175					0.01	0	0
chr4	944,210	rs34884217	A	C	Exonic	TMEM175	NSV	NM_032326	c.A194C	p.Q65P	0.06	0.09	0.1
chr4	945,017	rs200834686	A	G	Exonic	TMEM175	NSV	NM_032326	c.A313G	p.T105A	0.0034	0	0.001
chr4	945,059	rs767488180	C	T	Intronic	TMEM175					0.002	0	0.09
chr4	946,226	rs11552301	T	C	Exonic	TMEM175	SV	NM_032326	c.T450C	p.I150I	0.65	0.6	0.6
chr4	946,913	rs140188963	G	A	Intronic	TMEM175					0.01	0.002	0.009
chr4	946,974	rs2290405	G	A	Intronic	TMEM175					0.65	0.6	0.6
chr4	947,005	rs766495821	T	G	Exonic	TMEM175	NSV	NM_032326	c.T490G	p.F164V	0	0.005	0.07
chr4	947,062		C	T	Exonic	TMEM175	SNV_Stop	NM_032326	c.C547T	p.R183X	0.0017	0	0
chr4	947,180	rs183630281	C	T	Intronic	TMEM175					0.006	0.002	0.005
chr4	949,246	rs149676848	C	T	Exonic	TMEM175	SV	NM_032326	c.C681T	p.T227T	0.0017	0	0.0041
chr4	949,314	rs55790660	C	T	Intronic	TMEM175					0.01	0.005	0.005
chr4	949,537	rs778025081	C	A	Intronic	TMEM175					0.004	0	0.00006
chr4	949,625	rs34322727	C	T	Exonic	TMEM175	SV	NM_032326	c.C789T	p.A263A	0.006	0.015	0.0033
chr4	949,643	rs779176431	C	T	Exonic	TMEM175	SV	NM_032326	c.C807T	p.Y269Y	0	0.005	0.008
chr4	949,644	rs750645874	G	A	Exonic	TMEM175	NSV	NM_032326	c.G808A	p.A270T	0.004	0	0.00003
chr4	949,674		A	G	Exonic	TMEM175	NSV	NM_032326	c.A838G	p.I280V	0	0.005	0
chr4	949,854	rs144527015	C	T	Intronic	TMEM175					0.05	0.04	0.05
chr4	950,096	rs185191026	G	A	Intronic	TMEM175					0.01	0	0
chr4	950,159		C	T	Intronic	TMEM175					0.002	0	0
chr4	950,198		C	T	Intronic	TMEM175					0.0017	0	0
chr4	950,199	rs559887815	G	A	Intronic	TMEM175					0.0017	0	0.0002
chr4	950,256		A	T	Intronic	TMEM175					0.0017	0	0
chr4	950,286		A	G	Intronic	TMEM175					0.003	0	0
chr4	950,392		A	G	Intronic	TMEM175					0.0017	0	0
chr4	950,398	rs756095591	C	T	Intronic	TMEM175					0.0017	0	0
chr4	950,425	rs774718115	T	G	Intronic	TMEM175					0.003	0	0
chr4	950,483	rs187633990	C	T	Intronic	TMEM175					0	0.007	0.008
chr4	950,653	rs577228710	TGATATTCTC	T	Intronic	TMEM175					0.003	0	0
chr4	951,681	rs6854241	T	C	Exonic	TMEM175	SV	NM_032326	c.T912C	p.S304S	0	0.01	0.03
chr4	951,684	rs141810982	G	A	Exonic	TMEM175	SV	NM_032326	c.G915A	p.A305A	0.0017	0	0.0033
chr4	951,692	rs372100086	C	T	Exonic	TMEM175	NSV	NM_032326	c.C923T	p.P308L	0.0017	0	0.02
chr4	951,773	rs147762522	G	A	Exonic	TMEM175	NSV	NM_032326	c.G1004A	p.R335H	0.005	0.005	0.0022
chr4	951,791	rs80114247	T	C	Exonic	TMEM175	NSV	NM_032326	c.T1022C	p.M341T	0.035	0.019	0.03
chr4	951,812	rs147975675	C	T	Exonic	TMEM175	NSV	NM_032326	C1043T	p.S348L	0.004	0	0.001
chr4	951,858	rs145355643	G	A	Exonic	TMEM175	SV	NM_032326	c.G1089A	p.S363S	0.0017	0	0.0008
chr4	951,878	rs149156711	G	A	Exonic	TMEM175	NSV	NM_032326	c.G1109A	p.R370H	0.0017	0	0.0006
chr4	951,922		C	A	Exonic	TMEM175	NSV	NM_032326	c.C1153A	p.L385M	0.0017	0	0
chr4	951,924	rs767888769	G	C	Exonic	TMEM175	SV	NM_032326	c.G1340C	p.L385L	0.004	0	0.00001
chr4	951,947	rs34311866	T	C	Exonic	TMEM175	NSV	NM_032326	c.T1178C	p.M393T	0.24	0.18	0.2
chr4	951,982	rs75307864	C	G	Exonic	TMEM175	NSV	NM_032326	c.C1213G	p.L405V	0.0034	0	0.0038
chr4	952,000		T	C	Exonic	TMEM175	NSV	NM_032326	c.T1231C	p.F411L	0.0017	0	0
chr4	952,009	rs140597786	C	T	Exonic	TMEM175	NSV	NM_032326	c.C1240T	p.R414W	0.0017	0	0.0058
chr4	952,049	rs565504915	CCT	C	Exonic	TMEM175	FD	NM_032326	c.1281_1282del	p.P427fs	0.0024	0	0.0006
chr4	952,210	rs201314478	C	T	Exonic	TMEM175	NSV	NM_032326	c.C1441T	p.R481W	0.0036	0.005	0.0031
chr4	952,238	rs577438263	C	T	Exonic	TMEM175	NSV	NM_032326	c.C1469T	p.T490M	0	0.01	0.03
chr4	952,263		G	A	Exonic	TMEM175	SV	NM_032326	c.G1494A	p.Q498Q	0.0017	0	0
chr4	952,409	rs748483	A	G	UTR3	TMEM175		NM_032326	c.*125A > G		0.1	0.09	0.1

*POS* genomic position (hg19), *CHR* chromosome, *hg19* human genome build to which these variants are annotated, *SNP* single-nucleotide polymorphism, *dbSNP* reference number in SNP database, *ref seq* reference number of the gene transcript, *UTR* untranslated region, *AA change* amino acid change, *Nt Change* nucleotide change, *MAF Italian PD* allelic frequency in Italian PD patients, *MAF Italian CNT* allelic frequency in Italian controls, *NSV* non-synonymous variant, *SV* synonymous variant, *FD* frameshift deletion, *MAF* minor allele frequency, *MAF max in public datasets* highest allelic frequency annotated in public databases including 1000 Genomes Project (AFR. AMR. EAS. EUR. SAS), ExAC browser (NFE. AFR. SAS. EAS and AMR), ESP6500si-v2 (European American and African American population)

**Fig. 3:**
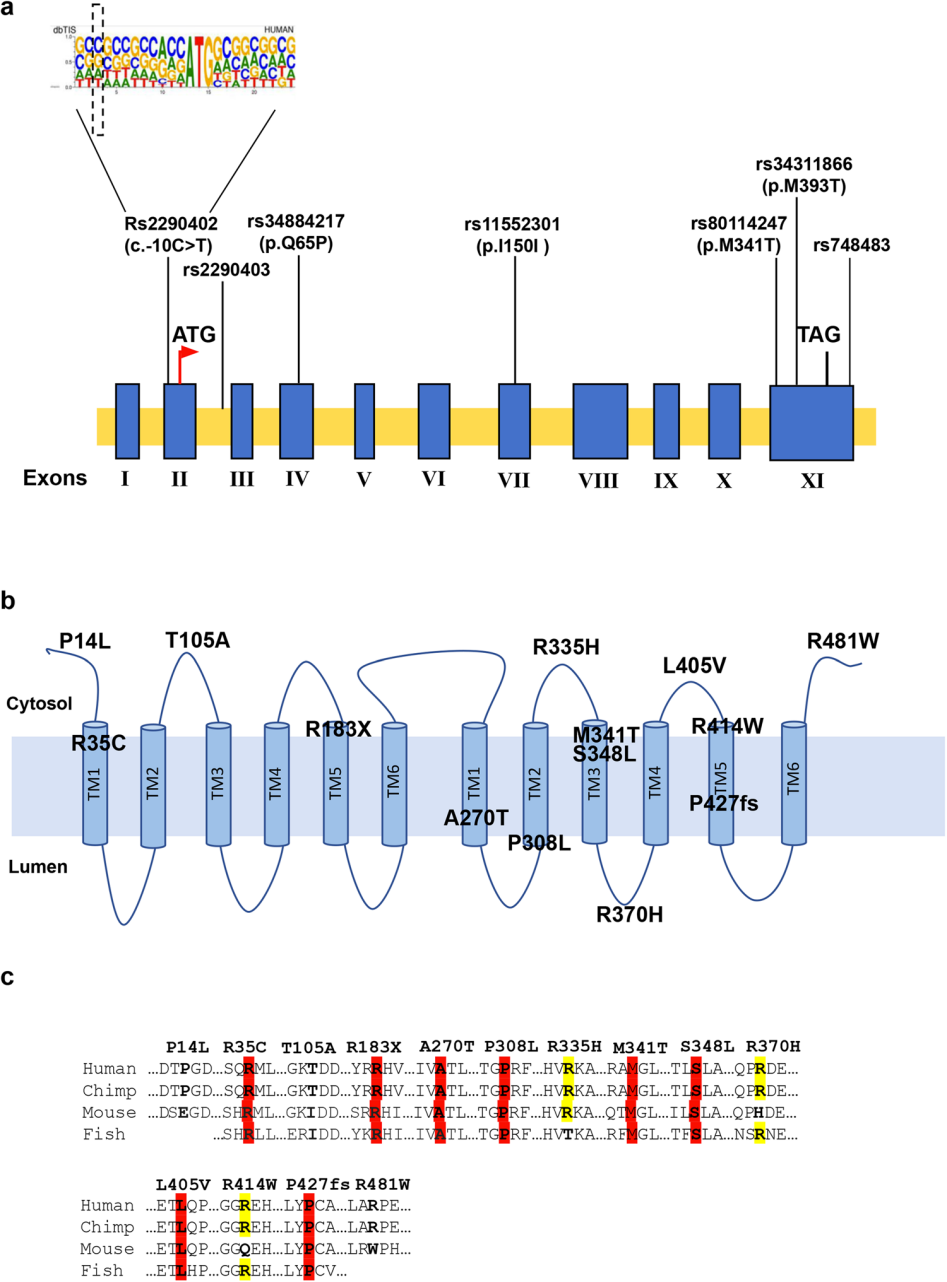
Graphical distribution of the *TMEM175* gene variants identified in Italian PD patients. **a** The 7 common variants identified in the *TMEM175* gene are reported on the exon/intron structure of the gene. Exons are shown as blue rectangles and introns as yellow bars. The start codon site (ATG) and the stop codon (TAG) are indicated. The human Kozak consensus sequence is reported in the upper part of the figure. **b** The 13 rare variants identified in the Italian PD patients are indicated on the graphical representation of the protein. TMEM175 consists in 6 trans-membrane motifs (TM1-TM6), each repeated twice. Five variants are exposed to the cytoplasm (p.P14L, p.T105A, p.R335H, p.L405V, and p.R481W), 8 variants are in the transmembrane motifs (p.R35C, p.R183X, p.A270T, p.P308L, p.M341T, p.S348L, p.R414W, and p.P427fs), and 1 is exposed to the lumen (p.R370H). **c** Eleven variants, including 10 rare variants and the p.M341T common variant, affect highly conserved residues among vertebrates (highlighted in red and yellow)

To perform a case–control association study, we selected 7 variants that were located throughout the gene (Fig. 3a), were annotated both in WES and NGS-TR dataset, and showed a minor allele frequency (MAF) > 0.01 in multiple genetic databases. Results are reported in Table 2. Four variants showed significant association with PD: rs2290402 (nucleotide position c.-10C > T; odds ratio (OR) (confidence interval (CI)) = 1.5 [1.12–2.04], *p* value (p) = 0.003), rs34884217 (c.A194C, amino acid position p.Q65P; OR [CI] = 0.67 [0.44–1.01], *p* = 0.03), rs80114247 (c.T1022C, p.M341T; OR [CI] = 1.84 [0.93–3.63], *p* = 0.05], and rs34311866 (c.T1178C, p.M393T; OR [CI] = 1.38 [1.02–1.87], *p* = 0.02). Interestingly, two of these variants (rs34884217 and rs34311866) have been already described as associated with PD in other populations [11, 12, 28], while rs2290402 and rs80114247 are novel variants never described before. The rs2290402 variant (transcript number NM_032326; c.-10C > T), which remained statistically significant after correction for multiple testing, is located upstream (in the 5′-direction) from the ATG start codon in the Kozak consensus sequence (Fig. 3a), suggesting a functional role in modulating protein translation. The rs80114247 variant (c.T1022C, p.M341T) affected a highly conserved residue located in the TM3-II transmembrane domain, suggesting a role in the channel activity (Fig. 3b, c).

**Table 2:** Case–control association analysis in the Italian cohort

Series	Position	PD	PD-HR	PD-het	PD-HM	CNT	CNT-HR	CNT-het	CNT-HM	P (11vs12 + 22)	OR	95%CI
rs2290402	c.-10C > T	447	235	182	30	300	188	102	10	**0.003**	1.5	1.12–2.04
rs2290403	Intron 2	401	196	166	39	300	131	133	36	0.09	0.81	0.6–1.09
rs34884217	p.Q65P	458	395	50	3	300	250	46	4	**0.03**	0.67	0.44–1.01
rs11552301	p.I150I	398	56	163	179	300	40	142	118	0.4	0.93	0.60–1.45
rs80114247	p.M341T	449	417	32	0	300	288	12	0	**0.05**	1.84	0.93–3.63
rs34311866	p.M393T	448	255	167	26	300	194	100	6	**0.02**	1.38	1.02–1.87
rs748483	c.*125A > G	288	230	52	6	300	247	53	0	0.25	1.17	0.77–1.77

*PD*, Parkinson’s disease; *HR*, homozygous reference; *het*, heterozygous; *HM*, homozygous mutated; *Cnt*: controls from TSI, Tuscan Italian population, and Neuromed (MNI) cohort genetic biobank; *p*, *p*-value calculated with Fisher Exact Probability Test; *OR*, odds ratio; *CI*, confidence interval. Statistically significant associations (*p* < 0.05) are highlighted in bold. Bonferroni correction for seven variants contrasts was applied, resulting in a corrected *α* = 0.007

The assessment of motor and non-motor PD symptoms revealed a significant association with Unified Parkinson’s Disease Rating and Non-motor Symptoms (UPDRS/NMS) scales for the rs2290402 variant (UPDRS: OR [CI] = 1.63 [0.99–2.63], *p* = 0.03; NMS: OR [CI] = 1.46 [0.98–2.20], *p* = 0.03). Overall data showed that the rs2290402 variant might influence the occurrence and the severity of the PD phenotype, although the contrast would not survive correction for multiple testing of five phenotypes (Table 3).

**Table 3:** Analysis of PD endophenotypes in 384 Italian PD patients

Series	Scores	rs2290402 (11)	rs2290402 (12 + 22)	P (11vs12 + 22)	OR	95% CI
AAO	**Age < 50 years**	39	31	0.5	1.02	0.6–1.72
	**Age ≥ 50 years**	173	141	Reference		
UPDRS	**Score < 30**	156	141	Reference		
	**Score ≥ 30**	56	31	**0.03**	1.63	0.99–2.63
MoCA	**Score < 26**	142	103	0.09	1.35	0.89–2.06
	**Score ≥ 26**	70	69	Reference		
LID	**No**	120	98	Reference		
	**Yes**	92	74	0.2	1.19	0.79–1.80
NMS	**Score ≤ 54**	103	100	Reference		
	**Score > 54**	109	72	**0.03**	1.46	0.98–2.20

*AAO* age at onset, *UPDRS* Unified Parkinson’s Disease Rating Scale Part III, *MoCA* Montreal Cognitive Assessment, *LID* Levodopa-induced dyskinesia, *NMS* non-motor symptoms, *p p*-value calculated with Fisher Exact Probability Test, *OR* odds ratio, *CI* confidence interval. Values in bold indicate statistically significant difference

### Analysis of Rare Detrimental Variants in *TMEM175* Gene

**Fig. 4:**
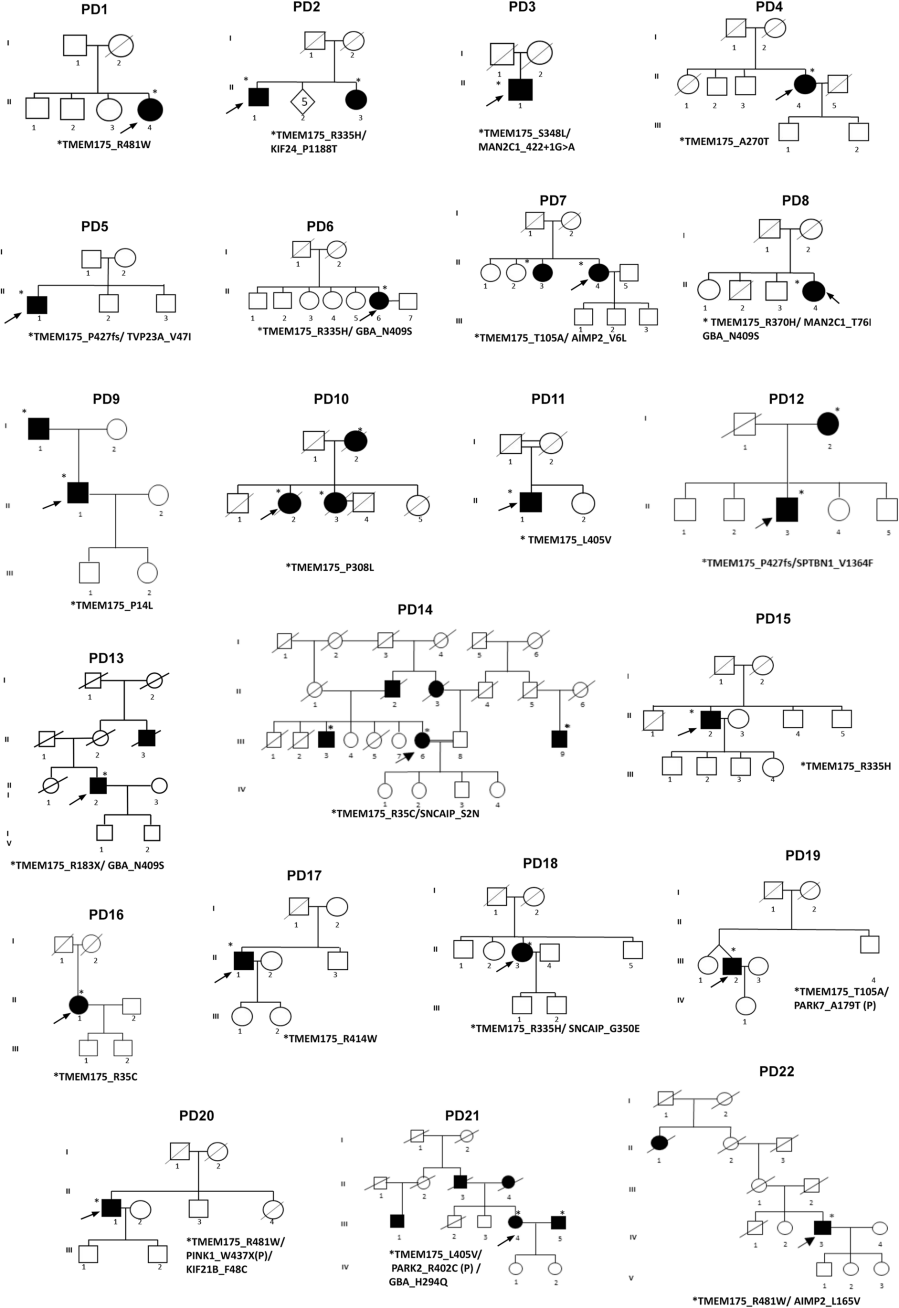
Graphical representation of the 22 Italian families carrying *TMEM175* mutations. Affected individuals are indicated with dark symbols. Patients who underwent WES analysis are shown with dark arrows. Additional family members from whom DNA was available are indicated with an asterisk. Only affected subjects were included in the segregation analysis. The rare deleterious variants reported in Table 4 were carried by the affected family members

Thirteen rare detrimental variants were identified in 22 Italian patients. All affected residues identified in PD patients occupied functionally important amino acid positions, and seven of them were highly conserved among vertebrates (Fig. 3b, c). Considering the polygenic inheritance associated with PD, disclosed in our previous study [8], we identified 4 patients carrying one mutation in *TMEM175* and one pathogenic mutation in *GBA* (p.N409S or H294Q); three patients carrying one mutation in *TMEM175* and one pathogenic mutation in *PARK2*, *PARK7*, and *PINK1* respectively; and 8 patients carrying one mutation in *TMEM175* and one rare deleterious variant in other PD candidate genes such as *AIMP2*, *KIF24*, *MAN2C1*, *SNCAIP*, *SPTBN1*, and *TVP23A*. Eight PD patients carried a single deleterious variant in *TMEM175* alone (Table 4, Fig. 4). The segregation analysis was performed only in 7 families (PD2, PD7, PD9, PD10, PD12, PD14, PD21) in which more PD patients, in the same family, were available for the genetic test (Fig. 4).

**Table 4:** Clinical characteristics of the Italian PD patients carrying deleterious variants in the *TMEM175* gene

Sample	IDFAM	Sex	Type	AAO	YD	UPDRS	LID	MoCA	NMS	Mutation *TMEM175*	Second and third mutation
PD1	37	F	SPD	65	5	10	Yes	25	10	R481W	
PD2	47	M	FPD	60	14	39	Yes	18	100	R335H	KIF24_P1188T
PD3	92	M	SPD	57	3	18	Yes	25	15	S348L	MAN2C1 422 + 1G > A
PD4	95	F	SPD	65	15	19	Yes	25	66	A270T	
PD5	96	F	SPD	44	6	6	No	30	0	P427fs	TVP23A_V47I
PD6	114	F	SPD	54	8	17	Yes	20	42	R335H	GBA_N409S
PD7	122	F	FPD	66	7	25	Yes	18	93	T105A	AIMP2_V6L
PD8	151	F	SPD	56	5	16	No	24	15	R370H	MAN2C1_T76P GBA_N409S
PD9	156	M	FPD	65	1	26	Yes	26	13	P14L	
PD10	186	F	FPD	65	na	10	Na	25	na	P308L	
PD11	196	M	SPD	65	5	18	No	17	90	L405V	
PD12	198	M	FPD	57	5	31	No	25	28	P427fs	SPTBN1_V1364F
PD13	215	M	FPD	54	19	21	Yes	18	88	R183X	GBA_N409S
PD14	262	F	FPD	67	3	10	No	25	48	R35C	SNCAIP_S2N
PD15	281	M	SPD	79	4	14	No	26	47	R335H	
PD16	297	F	SPD	63	13	19	Yes	20	54	R35C	
PD17	299	M	SPD	55	9	23	Yes	25	17	R414W	
PD18	307	F	SPD	44	11	19	Yes	27	54	R335H	SNCAIP_G350E
PD19	312	M	SPD	52	2	10	Yes	27	38	T105A	PARK7_A179T (P)
PD20	358	M	SPD	59	3	20	No	27	46	R481W	PINK1_W437X KIF21B_F48C
PD21	371	F	FPD	65	5	9	No	27	44	L405V	PARK2_R402C GBA_H294Q
PD22	385	M	FPD	54	17	22	No	24	46	R481W	AIMP2_L165V

*FPD* familial PD cases, *SPD* sporadic PD cases, *AAO* age at onset, *YD* years of the disease, *UPDRS* Unified Parkinson’s Disease Rating Scale Part III, *MoCA*, Montreal Cognitive Assessment, *LID* levodopa-induced dyskinesia, *NMS* non-motor symptoms

A complete description of all clinical data of the 22 patients is reported in Table 4; pedigrees are reported in Fig. 4. Mean age (± SD) at first symptoms was 59.5 ± 8.2 years (range 44–79 years). A higher age at onset (AAO) was found in patients carrying only the mutation in the *TMEM175* gene compared to patients also presenting mutations of other genes (62.5 ± 6.5 years vs 56.3 ± 7 years; *p* = 0.05). No difference in motor, non-motor, and cognitive (Montreal Cognitive Assessment (MoCA) score) symptoms were identified. Considering the high expression of *TMEM175* gene in microglia of human and mouse cerebral cortex, we investigated the presence of areas of cortical atrophy in our cohort of patients carrying detrimental mutations. Indeed, previous positron emission tomography (PET)/magnetic resonance imaging (MRI) studies have already largely investigated the relationship between neuroinflammation and gray matter alterations in Alzheimer’s disease [29–31]. However, more recently a negative correlation between an increased microglia activation and gray matter volume reduction has been demonstrated also in alpha-synucleinopathies such as PD [32]. Accordingly, in vitro evidences have suggested that neuronal release of aggregated alpha-synuclein in the central nervous system may activate microglia with production of proinflammatory mediators increasing the rate of neurodegeneration [32].

**Fig. 5:**
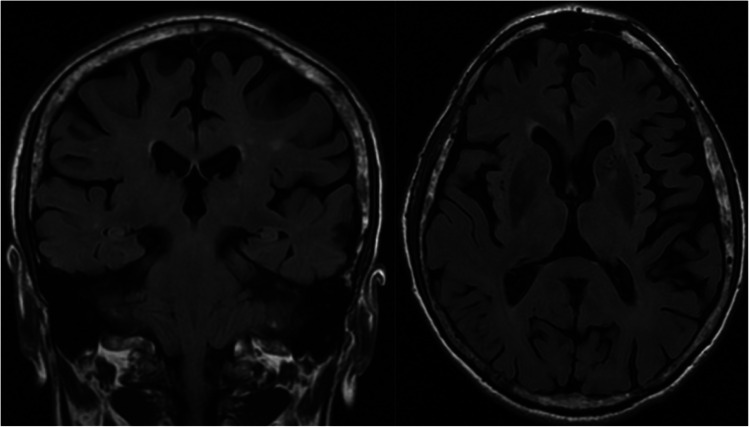
MRI analysis of PD patient. Three-dimensional T1-weighted images showing widespread cortical atrophy in a PD patient carrying the TMEM175 p.P14L mutation (coronal slice, left; axial slice, right)

Six patients underwent MRI in our Parkinson Centre. In five patients, MRI was performed in the first 2 years of the disease; in one patient carrying the p.P14L variant, MRI performed at 5-year follow-up showed widespread cortical atrophy (Fig. 5).

Overall, these data did not permit us to conclude that the mutations in the *TMEM175* gene might be sufficient to cause the disease; however, the co-inheritance of mutations in other PD genes anticipated the clinical onset of the disease of about 6 years.

### Functional Analysis of TMEM175 Deleterious Variants

**Fig. 6:**
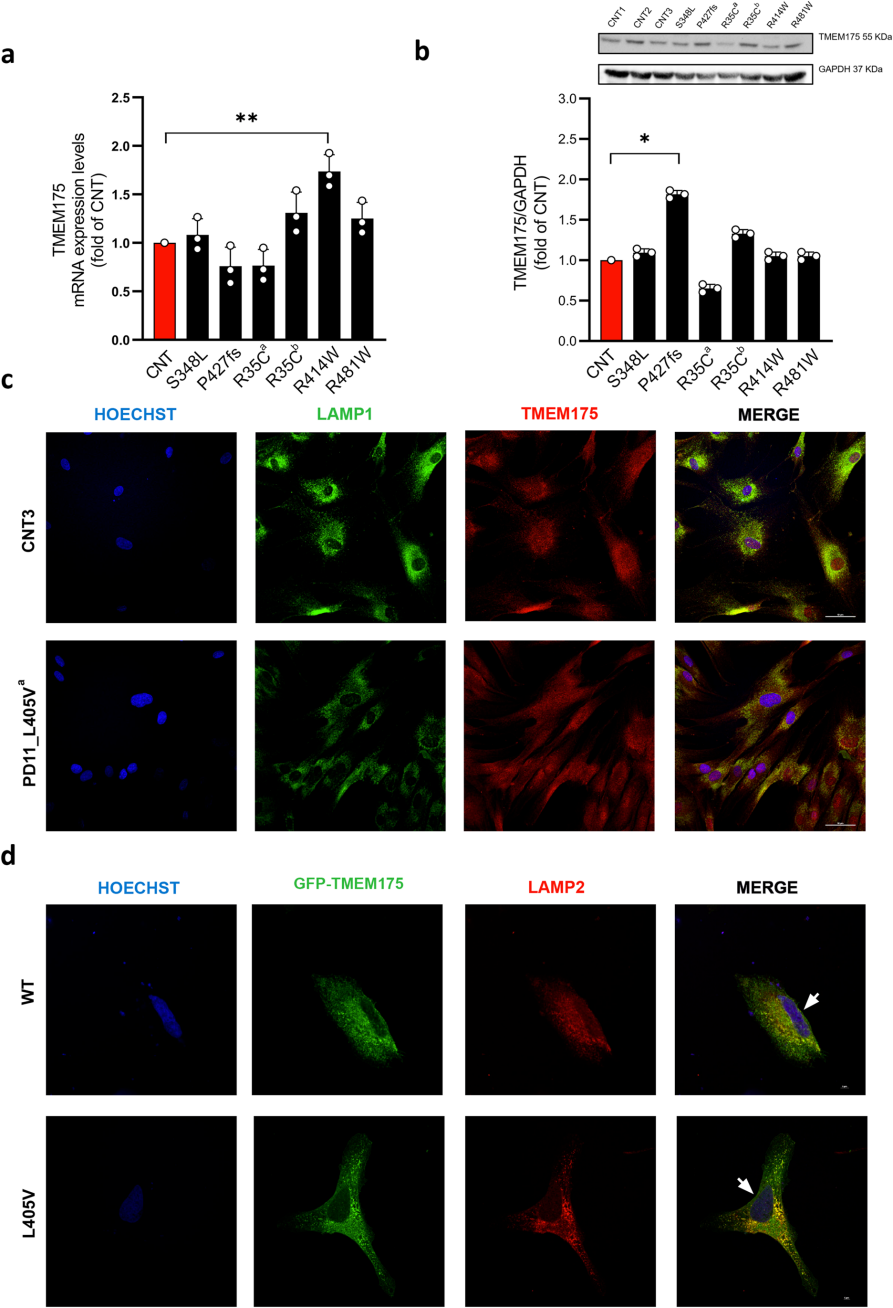
Expression analyses of TMEM175 in hDF of PD patients and healthy subjects. **a** qPCR and **b** western blot experiments. A nonuniform gene expression pattern was observed between the two groups (PD patients, black bar in the graph; healthy subjects, red bar in the graph). The mutations p.S348L, p.P427fs, p.R35C^a^, p.R35C^b^, p.R414W, and p.R481W correspond to the dermal fibroblasts of the patients PD3, PD5, PD14, PD16, PD17, and PD22, respectively (Table 4). Data were normalized to GAPDH and expressed as absolute values with SD calculated from three biological replicates. A significant increase in protein levels was observed in PD5 carrying the p.P427fs mutation. **c** and **d** Immunofluorescence experiments to visualize the sub-cellular localization of the TMEM175 protein. **c** Human dermal fibroblasts (hDF) of the healthy subject (CNT3) and of the PD11 patient carrying the p.L405 mutation. **d** HeLa cells transfected with the wild-type (WT) or the mutant (L405V) TMEM175 protein GFP-tagged. Hoechst H3570 (blue) was used to detect nuclei. **c** In hDF the endogenous TMEM175 protein was stained with the anti-TMEM175/CY3 (red), and the lysosomal markers LAMP1 was stained using anti-LAMP1/488 (green). **d** In HeLa cells, the exogenous GFP-tagged TMEM175 protein was in green, and the lysosomal marker LAMP2 was stained using anti-LAMP2/CY3 (red). Both endogenous TMEM175 protein and exogenous GFP-tagged TMEM175 protein are co-localized with lysosomal markers. White arrows in **d** indicate the localization of the GFP-tagged TMEM175 protein into the plasma membrane. Images were acquired by Nikon Confocal Microscope A1R, and scale bars correspond to **c** 50 μm and **d** 5 μm

TMEM175 is an endolysosomal K^+^ channel that regulates lysosome pH stability and K^+^ conductance [15]. We evaluated the impact of the most detrimental variants identified in the *TMEM175* gene in Italian PD patients on mRNA and protein stability and sub-cellular localization in dermal fibroblasts (hDF) derived from PD patients and healthy subjects (Fig. 6a–c). We observed a nonuniform gene expression pattern between the two groups. In particular, a significant increase in the protein amount was observed in PD5 carrying the p.P427fs mutation (Fig. 6b), suggesting that this mutated protein might accumulate into the cell. Interestingly, reduced amount of TMEM175 protein was observed in hDFs derived from PD14 carrying the p.R35C mutation co-inherited with the SNCAIP_S2N mutation, compared to the expression observed in PD16 carrying the p.R35C mutation alone, suggesting that the expression of TMEM175 might be perturbed by the polygenic variant burden (Fig. 6b). A co-immunostaining with the lysosomal marker, LAMP1, was observed in patient and control cells, suggesting that these mutations did not affect protein folding and localization (Fig. 6c, Fig. S1).

However, as the analyzed patients were heterozygous for *TMEM175* mutations, we were not able to clearly discriminate between reference and mutant alleles. We also generated a panel of GFP-tagged wt and mutant proteins (Fig. 3c) and demonstrated that mutant proteins were able to translocate to lysosomal membranes in HeLa cells (Fig. 6d, Fig. S2). However, we also observed intense staining signal into the cytoplasm and faint staining signal on plasma membrane, suggesting that the excess protein, produced by the construct, remained free in the cytoplasm or translocated into plasma membrane (Fig. 6d, Fig. S2).

**Fig. 7:**
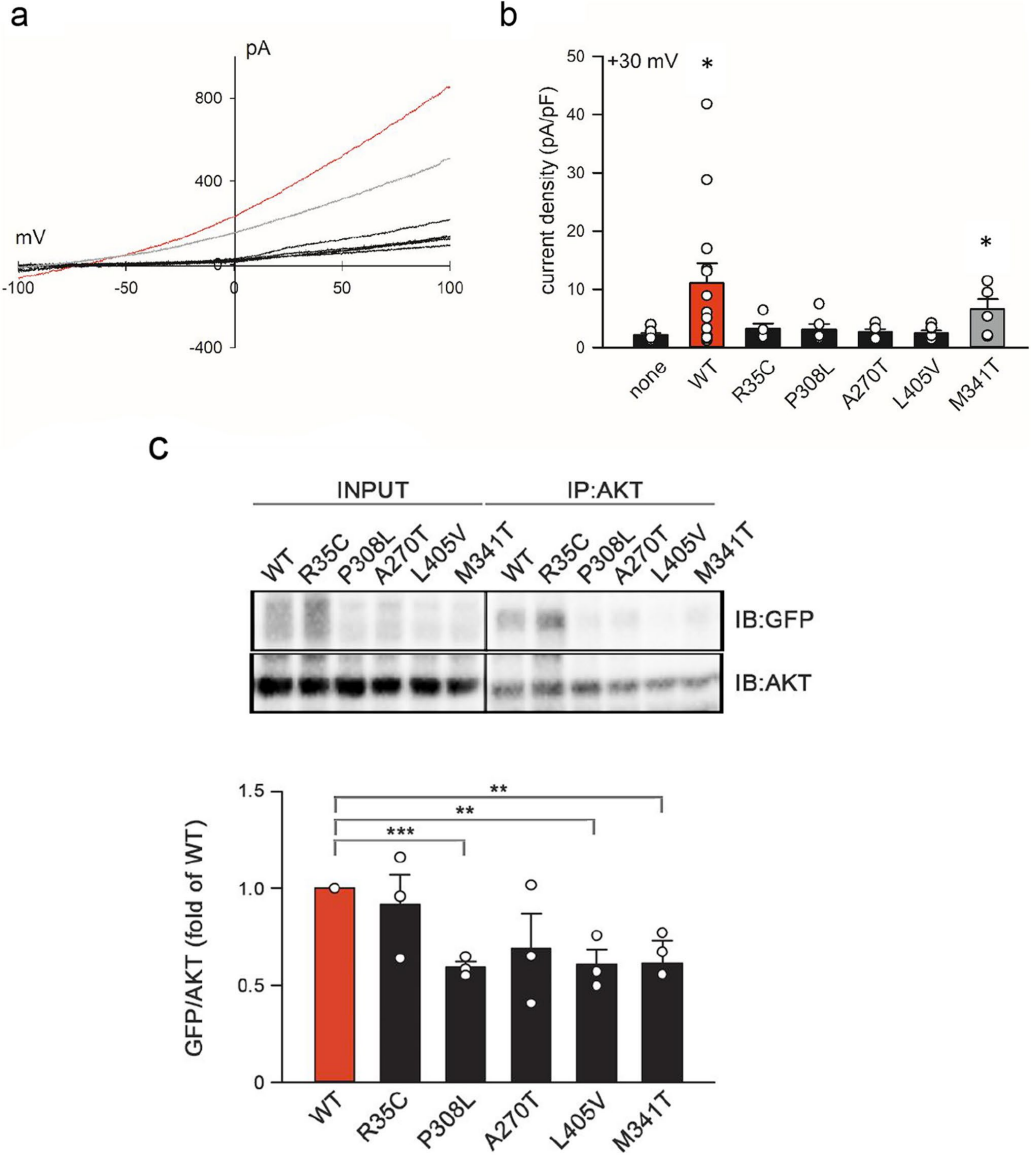
Mutations in TMEM175 alter channel activity. **a** Typical I-V relation obtained by applying a single voltage ramp (from − 100 to + 100 mV, 200 ms) on a representative HEK293 cell transfected with wt TMEM175-GFP-fused construct (red line), TMEM175-M341T polymorphic variant (gray line), and TMEM175 R35C, P308L, A270T, and L405V rare deleterious variants (black line). Note the increase in the current in cell overexpressing the wt plasmid. **b** Histogram representing the mean current density measured at + 30 mV in different experimental conditions, as indicated. Note the increase in current density in cells overexpressing the wt TMEM175 protein compared to non-transfected cells (*n* = 8 and *n* = 13 biological replicates, respectively). Current density measured in cells transfected with the mutant proteins TMEM175 R35C, P308L, A270T, and L405V (*n* = 5, *n* = 6, *n* = 6, *n* = 7 biological replicates, respectively) was similar to that measured in non-transfected cells, while the polymorphic variant TMEM175 M341T showed an increase of the K ^+^ current across the plasma membrane that was intermediate between that detected with the wt and mutant protein (*n* = 6 biological replicates). Data (mean ± SEM) were analyzed with Student’s t-test and plotted as histogram representation. (*) indicates a *p* value < 0.05. **c** On the top representative immunoblotting of anti-GFP in immunoprecipitated protein with anti-Akt in MN9D cells expressing GFP-conjugated TMEM175 wt and mutant proteins. In the left, total proteins blotted for input control. On the bottom, representative histogram of the fold of wt densitometric ratio between the anti-GFP and anti-Akt blots (*n* = 3 biological replicates). Note the loss of binding between Akt and the mutant TMEM175 alleles P308L, L405V, and M341T expressed in stable cell line. The mean ± SEM of three biological replicates were analyzed with Student’s t-test. Data were plotted as histogram representation. (**) indicates a *p* value < 0.01, (***) indicates a *p* value < 0.001

Next, we investigated the functional properties of the mutant channels in HEK293 cells. In transfected HEK293 cells, the GFP-tagged TMEM175 protein was able to translocate into the plasma membrane (data not shown), as also demonstrated in a previous study [15]. Electrophysiological measurements were performed in the perforated patch-clamp configuration, avoiding cell dialysis [33]. Under this condition, during a stimulation protocol consisting of voltage ramps from − 100 to + 100 mV applied every 2 s, overexpression of the wt channel induced a clear increase in transmembrane current, as exemplified in Fig. 7a. Current density measured at + 30 mV changed from a mean value of 2.15 ± 0.34 pA/pF in non-transfected cells to a fivefold increased value of 11.08 ± 3.38 pA/pF (*p* = 0.027) in cells overexpressing the wt protein (Fig. 7b). The mutant channels (p.R35C, p.P308L, p.A270T, p.L405V) showed no significant current increase compared to non-transfected cells (Fig. 7b; 3.25 ± 0.84 pA/pF *n* = 5; 3.11 ± 0.94 pA/pF, 2.75 ± 0.44 pA/pF, and 2.54 ± 0.41 pA/pF, respectively), suggesting that these variants might affect channel activity. Interestingly, the polymorphic variant p.M341T, associated with PD in the case–control analysis, showed an increase of the K^+^ current across the plasma membrane (6.63 ± 1.67 pA/pF, *p* = 0.043), which was intermediate between that detected with the wt and mutant proteins (Fig. 7b), indicating that this change might partially affect the functional properties of the channel.

Furthermore, we investigated if some of the identified changes affected the binding with the regulatory factor protein kinase B (Akt) [19]. The interaction was investigated in MN9D cells with a stable expression of GFP-tagged wt or mutant proteins. A significant reduction in Akt-binding affinity was observed for the p.P308L, p.L405V, and p.M341T mutations (*p* < 0.001, *p* = 0.007, *p* = 0.006, respectively) (Fig. 7c), located across the TM2II-TM4II domains of TMEM175 (Fig. 3c).

### TMEM175 Mutations Affect the Expression of Markers Involved in the Autophagy-Lysosomal Pathway and Unfolded Protein Response (UPR)

**Fig. 8:**
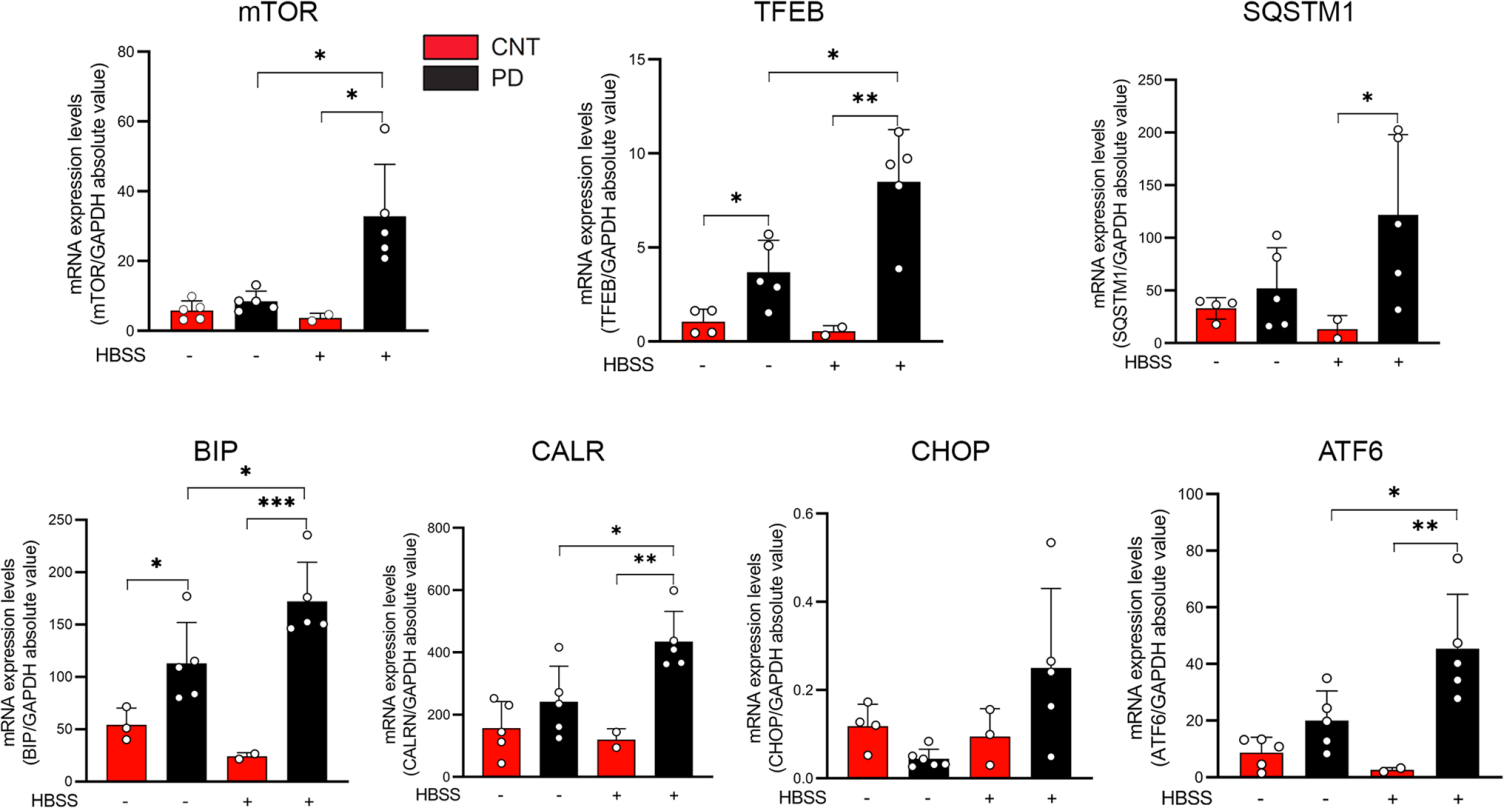
TMEM175 mutations affect the expression of markers involved in the autophagy-lysosomal pathway and unfolded protein response (UPR). qPCR experiments were performed in human dermal fibroblasts, under basal conditions (HBSS −) and under serum starvation (1 h) (HBSS +) to examine the expression of genes involved in the surveillance of autophagy-lysosomal and URP pathways. In the graph, dermal fibroblasts of healthy subjects (mean value of 5 healthy subject-derived cells: CNT1-CNT5) are indicated as red bars and dermal fibroblasts of PD patients (mean value of 6 patient-derived cells: PD3, PD5, PD14, PD16, PD17, and PD22 carrying the mutations p.S348L, p.P427fs, p.R35C^a^, p.R35C.^b^, p.R414W, and p.R481W respectively (Table 4) are indicated as black bars. The mean ± SD of three biological replicates was analyzed with Student’s t-test. Data were plotted as histogram representation. (*) indicates a *p* value < 0.05, (**) indicates a *p* value < 0.01, (***) indicates a *p* value < 0.001

**Fig. 9:**
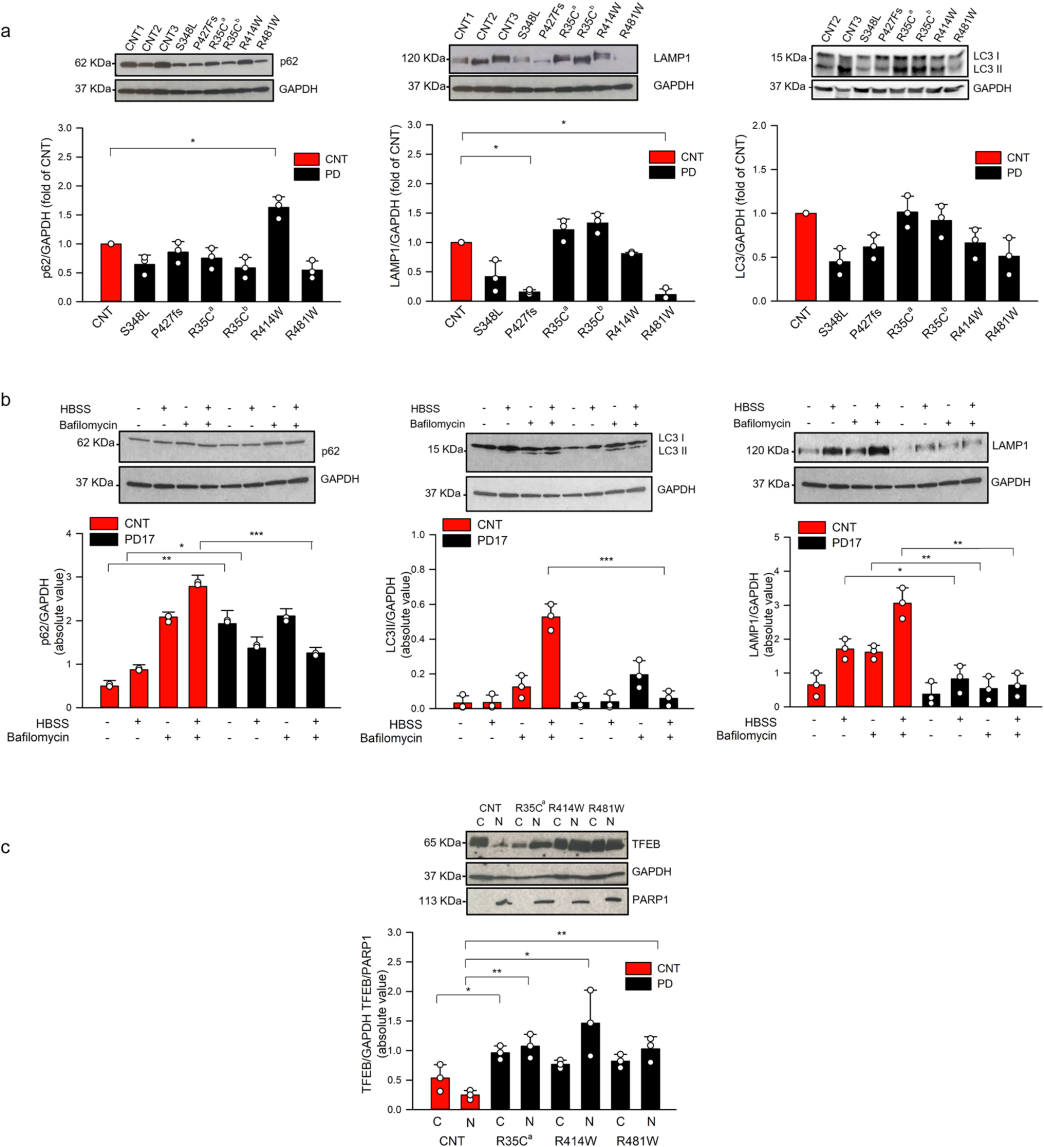
Autophagy-lysosomal pathway was defective in patient-derived fibroblasts. **a** Cells were harvested for 1 h, and the transformation of LC3 I into LC3 II and the expression of p62 and LAMP1 were detected by WB. Patient-derived cells show a reduced expression of LC3 II and LAMP1 compared to healthy subjects. The mean ± SD of three biological replicates was analyzed with Student’s t-test. Data were plotted as histogram representation: healthy subjects (mean value of 3 healthy subject-derived cells) are indicated as red bars, and patients are indicated as black bars. The mutations p.S348L, p.P427fs, p.R35C^a^, p.R35C^b^, p.R414W, and p.R481W correspond to the dermal fibroblasts of the patients PD3, PD5, PD14, PD16, PD17, and PD22, respectively (Table 4). (*) indicates a *p* value < 0.05, (**) indicates a *p* value < 0.01, and (***) indicates a *p* value < 0.001. **b** Cells were harvested for 2 h in HBSS w/wo 100 mM bafilomycin. Expression of LC3 I/LC3 II, p62, and LAMP1 was detected by WB. PD17-derived cells showed a reduced expression of p62, LC3 II, and LAMP1. Note that PD17 is a heterozygous carrier of TMEM175-R414W alone. **c** The translocation of TFEB into the nucleus was detected by WB. Patient-derived cells show a significant increase of expression of TFEB protein in the nucleus compared to healthy subjects

**Fig. 10:**
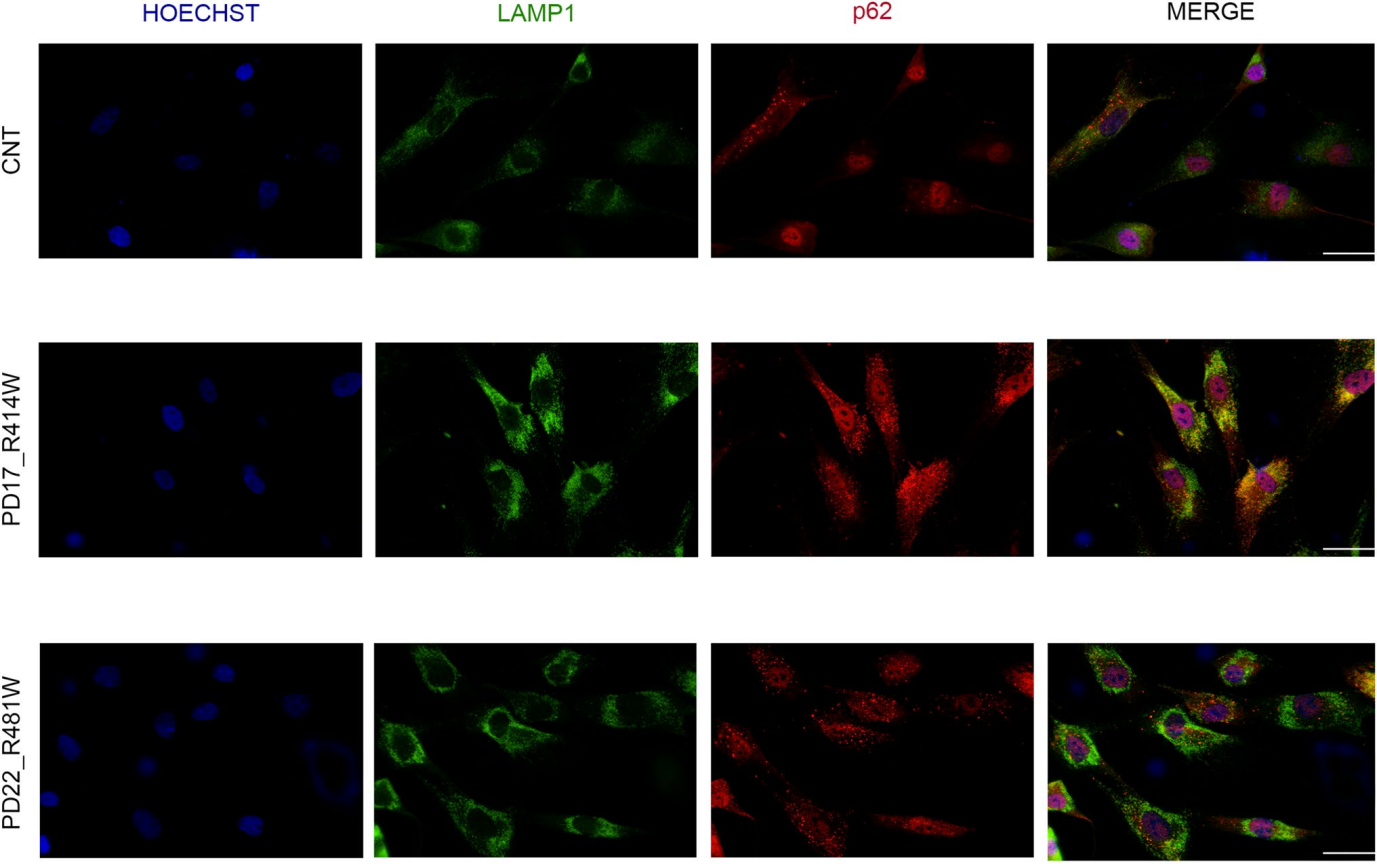
Immuno-localization of LAMP1 and p62 in patient-derived fibroblasts. The lysosomal marker, LAMP1, is in green, whereas the autophagic marker, p62, is in red. Under stress conditions, patient-derived fibroblasts displayed an increased number, size, and intensity of p62 puncta (white arrow in the lower panel) compared to healthy subject-derived cells. Hoechst H3570 (blue) was used to detect nuclei. Slides were visualized with Nikon Eclipse Ni-E 60 × magnification, and scale bars correspond to 50 μm

Quantitative PCR analysis showed, under stress conditions (1-h serum starvation), a significant increase in the expression of the mRNA of the autophagy-lysosomal markers, TFEB, mTOR, and SQSTM1, and the UPR markers, BIP, ATF6, and CALR, in patient-derived fibroblasts (PD3, PD5, PD14, PD16, PD17, PD22) compared to fibroblasts of healthy-unrelated subjects (CNT1-CNT5) (Fig. 8). A significant increase in the mRNA expression of TFEB and BIP was also observed under basal conditions (Fig. 8). A reduced expression of LC3 II and LAMP1 proteins was observed in PD3, PD5, PD17, and PD22 (Fig. 9a), and a significant increase in the expression of TFEB protein was observed in the cell nucleus in patients PD14, PD17, and PD22 (Fig. 9c). These data were suggestive of a defective autophagy in PD patients, either mutated in *TMEM175* alone (PD17) or carrying two pathogenic mutations, one in *TMEM175* and one in another PD gene (PD3, PD5, PD14, and PD22) (Table 4). A significant reduction of p62, LC3 II, and LAMP1 was also observed in PD17 cells (p.R414W-TMEM175), under bafilomycin treatment, further supporting autophagy impairment and a causal role for this mutation (Fig. 9b). p62 regulates the formation of protein aggregates and is removed by autophagy. Accumulation of p62-positive aggregates in cells and tissues are among the best-known characteristics of autophagy-deficiency; in fact, an increase or decrease in the amounts of p62 protein and aggregates can reflect a change in autophagic activity [34]. In patient-derived cells carrying *TMEM175* mutations we observed, under stress conditions, a larger number of p62 punctate signals that were suggestive of impaired autophagy, compared to healthy subjects (Fig. 10, Fig. S3).

**Fig. 11:**
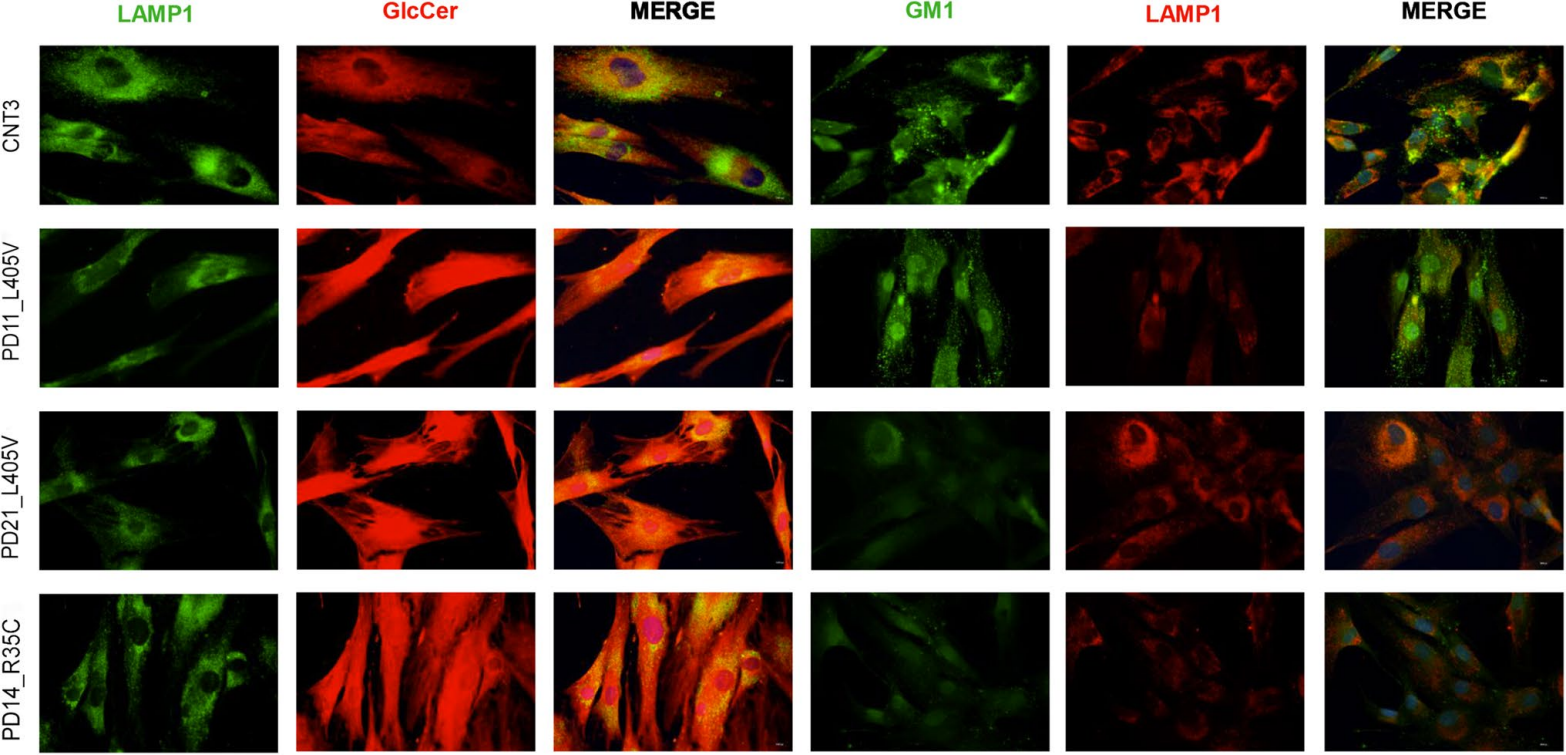
Immuno-localization of glucosylceramide (GlcCer) and ganglioside GM1 in patient-derived fibroblasts. LAMP1 is shown green when associated with Glc-Cer (in red) and is shown in red when associated with GM1 that was detected with Cholera toxin subunit B (CT-B)/488. Nuclei were stained with Hoechst. Slides were visualized with Nikon Eclipse Ni-E 60 × magnification. A strong intense signal was observed for GlcCer in patient-derived cells. GM1 was reduced in patients compared to control cells

Finally, we investigated whether *TMEM175* mutations might affect the amount and distribution of glucosylceramide (GlcCer), which is cleaved into glucose and ceramide by the lysosomal enzyme glucocerebrosidase (GBA), as well as the expression of ganglioside GM1, which is reduced in PD patients [35]. Immunofluorescent experiments in hDF showed staining of GlcCer and GM1 in the cytoplasm and lysosomes. Higher levels of GlcCer, together with reduced amount of GM1, were observed in PD patients compared to healthy controls (Fig. 11). These changes were prominent in patients PD21 and PD14 carrying three mutations (TMEM175 L405V/PARK2 R402C/GBA H294Q) and two mutations (TMEM175 R35C/SNCAIP S2N), respectively (Fig. 11). Interestingly for PD11, which carried the TMEM175 L405V mutation alone, no difference in GM1, compared to healthy subjects, was observed (Fig. 11).

## Discussion

To date, the genetic diagnosis of PD is still challenging due to the high genetic heterogeneity associated with the disease and the difficulty in interpreting results of genetic testing. Genetic characterization may pave the way to tailored treatments targeting different pathophysiological mechanisms in individual PD patients [36].

In the current study, we examined the role of common and rare variants of the *TMEM175* gene in Italian PD patients through the analysis of next-generation sequencing data followed by association tests.

We found a statistically significant association between PD and the common *TMEM175* variant rs2290402 (c.-10C > T; *p* = 0.003) and nominal associations (*p* < 0.05) between PD and the other variants, rs34884217 (c.A194C, p.Q65P), rs80114247 (c.T1022C, p.M341T), and rs34311866 (c.T1178C, p.M393T) (Table 2). Interestingly, the most associated variant, in our cohort of patients, was observed in the Kozak consensus sequence, 10 nucleotides upstream of the ATG start codon. The presence of the T allele predicted the ATG start site with a score 8% lower than the C allele, by using the ATGpr software (https://atgpr.dbcls.jp). This suggests the association of PD with a putative regulatory variant, affecting the level of the translated protein. Further association studies, performed on a higher number of subjects and targeting both intronic regions and other potentially regulatory regions outside the 5′ and 3′ UTRs will be necessary to characterize any association between PD and other potential additional *TMEM175* variants.

To establish whether rare and highly penetrant variants of *TMEM175* were causal mutations, we analyzed the segregation of the selected changes in PD families in which multiple patients were available. We identified 13 rare variants in 22 PD patients. These included 1 frameshift, 1 stop codon, and 11 missense amino acid changes, 8 of them affecting highly conserved amino acid residues (Fig. 3c).

In several patients, the same *TMEM175* variant was either present as single causal mutation or co-inherited with mutations in other PD genes, and the presence of multiple mutations in PD genes is associated with earlier onset of the disease.

From a survey of the literature, we observed that some of the identified changes were in important domains of the protein. In particular, the p.R35C change is in the RxxxFSD motif, which is important for channel folding [15], p.A270T is in the TM1-II domain, important for the selectivity of K^+^ permeability [15], p.P308L is in the TM1II-TM2II linker, which protrudes outwards from the main body of the TM domain and is the entrance space of the channel pore [15], and p.M341T and p.S348L are in the TM3-II domain.

In the light of that, we investigated whether some of the identified mutations might have any functional consequences. To this regard, the electrophysiological analysis revealed a significant effect on channel opening probability for all selected mutations (p.R35C, p.A270T, p.P308L, p.L405V), while the effect, mediated by the common variant p.M341T, was less dramatic.

A recent study demonstrated that TMEM175 channel is activated by growth factors and gated by Akt [19]. The authors showed that Akt binds the TM2-TM3 domains of repeat II, and this complex is required for the channel to remain open after activation. Our results are coherent with these observations and revealed a significant reduction of Akt-TMEM175 binding affinity for the mutant proteins p.P308L, p.L405V, and p.M341T, which are in the TM2-TM3 domains of repeat II. These findings confirmed the key role of these amino acidic residues in the TMEM175/Akt interaction [19].

Considering the important role of the lysosomal-autophagic pathway in the pathogenesis of PD [37–39], we analyzed this pathway in patient-derived fibroblasts heterozygous for the mutant proteins p.R35C, p.S348L, p.R414W, p.P427fs, and p.R481W. We revealed a defective expression of different proteins involved in this pathway, including p62, LC3I/II, LAMP1, and TFEB in both basal and upon stress conditions. In particular, we found increased expression of TFEB at both mRNA and protein level and a massive TFEB translocation from a cytosolic location to the nucleus. This observation provided evidence for endogenous activation of TFEB as an adaptive response to lysosomal stress [37]. These findings were in line with other studies which demonstrated the requirement of TMEM175 for the maintenance of the physiological functions of lysosomes [15, 16, 19]. In fact, it is well demonstrated that TMEM175 depletion alters lysosome pH and leads to abnormal organelle fusion during autophagy [16, 19].

Moreover, we observed in these patients the activation of the UPR markers BIP, ATF6, and CALR, upon stress condition. This data are consistent with the results of other studies showing that sustained endoplasmic reticulum (ER) stress and dysfunction of autophagy are closely related in PD pathology [40].

Additional evidence of a possible impairment in lysosomal activity emerged by the analysis of glycosphingolipids in human fibroblast from PD patients.

A number of studies indicate that TMEM175 deficiency is associated with a decreased GBA activity and with the accumulation of GlcCer within lysosomes. This leads to impaired lysosomal protein degradation and increased exosomal release of alpha-synuclein [17, 41–43]. On the other hand, reduced levels of GM1, observed in both brain and peripheral tissues of PD patients, has been reported to contribute to the pathogenesis of the disease [29, 44]. Our data are in line with these observations and suggest an impairment of glycosphingolipid metabolic pathways in PD subjects, carrying *TMEM175* mutations, possibly due to defective endomembrane systems.

Taken together, these data suggest that the studied variants (p.R35C, p.A270T, p.P308L, p.M341T, p.S348L, p. L405V, p.R414W, p.P427fs, p.R481W) might affect important domains of the protein and alter lysosome functioning, suggesting a pathogenic relevance. Among the other identified variants, p.R183X is a truncating mutation that might affect protein function. We also performed in silico predictions of the variants p.T105A, p.R335H, and p.R370H with Dynamut software that allows assessing the impact of mutations on protein stability and dynamics [45]. We showed an alteration of the interatomic interactions and of the vibrational entropy energy between wt and mutant proteins, suggesting a possible functional role for these changes (Fig. S4).

Interestingly, the nine highly penetrant pathogenic mutations (p.R35C, p. R183X, p.A270T, p.P308L, p.S348L, p. L405V, p.R414W, p.P427fs, p.R481W) identified in *TMEM175* gene were present in 14 unrelated PD patients who represent the 3.5% of the entire MNI cohort. This percentage increases at 5.5% if we also consider those patients carrying the three mutations p.T105A, p.R335H, and p.R370H, predicted as deleterious by in silico analysis. This data might be very promising for the genetic diagnosis of PD if we consider that the major causative event of late onset PD is the p.G2019S mutation in *LRRK2* [46], which is present in our cohort in 8 out of 400 PD patients (2%) [8]. Therefore, in our opinion, inserting the genetic analysis of *TMEM175* gene in the diagnostic protocols of late-onset PD should be recommended; this could allow to diagnose the disease in a larger number of patients.

Another interesting finding emerging from our data is the demonstration that, in human and mouse brain, *TMEM175* is highly expressed in mdDA neurons of the SNpc and in microglia in the cerebral cortex. MRI carried out in one patient carrying the p.P14L mutation located in the signal peptide of TMEM175 showed intense areas of widespread cortical atrophy. This evidence paves the way to an in-depth investigation of a larger cohort of patients to understand if mutations in *TMEM175* may be responsible for a more severe phenotype characterized by cortical atrophy, as a result of neuroinflammation with ensuing neurodegeneration.

In recent years, inflammatory processes have emerged as prominent contributors to the pathogenesis of PD. A large body of evidence suggests that microglial cells may either protect or damage surrounding neurons depending on their phenotype. A proinflammatory, damaging, microglial phenotype is consistently observed in the PD brain [47]. It is well demonstrated that PD genes, including between others, *SNCA* and *LRRK2*, are expressed in microglial cells [48], and dominant mutations in these genes affect microglial function by promoting neuroinflammation via activation of microglia and inflammatory signaling pathways [49–51]. Moreover, it is well described that autophagy is involved in the regulation of the inflammatory status of microglia [52, 53] and may have a role in the pathophysiology of inflammation [54].

## Conclusions

Our results highlight the importance of performing fine mapping of common and rare variants of PD genes to obtain a comprehensive genetic contribution on pathogenesis of the disease and to ameliorate the genetic tests. Furthermore, the study identifies functionally important amino acid residues in TMEM175 that cause the disease. These findings will pave the way to facilitate and accelerate the identification of molecular targets capable of modulating the channel’s activity.

## Supplementary Information

Below is the link to the electronic supplementary material.

Supplementary file1 (DOCX 21 KB)

Supplementary file2 (PNG 5354 KB)

Supplementary file3 (PNG 2468 KB)

Supplementary file4 (PNG 2544 KB)

Supplementary file5 (PNG 1249 KB)

## Data Availability

The datasets used and/or analyzed during the current study are available from the corresponding author on reasonable request.

## Abbreviations

PD: Parkinson’s disease
LOPD: Late-onset Parkinson’s disease
GWAS: Genome-wide association studies
mdDA: Mesencephalic-diencephalic dopaminergic
SNpc: Substantia nigra pars compacta
WES: Whole exome sequencing
MNI: Mediterranean Neurological Institute
NGS: Next-generation sequencing
TR: Targeted resequencing
SNVs: Single-nucleotide variants
MAF: Minor allele frequency
CADD: Combined annotation-dependent depletion
TSI: Tuscan Italians
qPCR: Quantitative PCR
FPD: Familial PD
SPD: Sporadic PD
ER: Endoplasmic reticulum
