# Supplementary material for: Common and Rare Variants in TMEM175 Gene Concur to the Pathogenesis of Parkinson’s Disease in Italian Patients
Source: Mol Neurobiol. 2023 Jan 7;60(4):2150–73. doi: 10.1007/s12035-022-03203-9 (PMC9984355; doi:10.1007/s12035-022-03203-9)
Supplement: Supplementary file 1 — Supplementary file1 (DOCX 21 KB) [file 12035_2022_3203_MOESM1_ESM.docx]

Common and rare variants in TMEM175 gene concur to the pathogenesis of Parkinson’s disease in Italian patients

Nicole Piera Palomba ^1,#^, Giorgio Fortunato ^2,3,#^, Giuseppe Pepe ^1^, Nicola Modugno ^1^, Sara Pietracupa ^1^, Immacolata Damiano ^2^, Giada Mascio ^1^, Federica Carrillo ^2^, Laura Ianiro ^1^, Katiuscia Martinello ^1^, Viola Volpato ^4^, Vincenzo Desiato ^1^, Riccardo Acri ^1^, Marianna Storto^1^, Ferdinando Nicoletti ^1,5^, Caleb Webber ^4,6^, Sergio Fucile ^1,5^, Vittorio Magione ^1^, and Teresa Esposito ^1,2,^*

## Supporting information

**Legend to Supplementary figures**

**Fig. S1** Immuno-localization of TMEM175 and LAMP1 in human dermal fibroblasts. TMEM175 protein was stained using anti-TMEM175/CY3, LAMP1 lysosomal marker was stained using anti-LAMP1/488. Nuclei were stained with Hoechst. Intense co-staining with LAMP1 was observed for wt and mutant proteins, suggesting that the mutations did not affect the localization of the protein on lysosome. Slides were visualized with Nikon Confocal Microscope A1R at 60x magnification.

**Fig. S2** Immuno-localization of TMEM175 wt –GFP tagged and mutant (TMEM175-mut-GFP) and LAMP2 lysosomal marker in HeLa cells. GFP-tagged wild-type and mutant proteins were visualized as green spots, LAMP2 was stained using anti-LAMP2 /CY3. TMEM175 localyzed on lysosomal membrane in HeLa cells (Figure 5d, Figure S2). However, we also observed Intense staining signals were observed into the lysosome (co-staining with LAMP2), into the cytoplasm and faint staining signal on plasma membrane. No difference was observed between wt and mutant proteins. Nuclei were stained with Hoechst. Slides were visualized with Nikon Confocal Microscope A1R at 60x magnification.

**Fig. S3** Immuno-localization of LAMP1 and p62 in patient-derived fibroblast. LAMP1 was stained with anti-LAMP1 /488 and p62 autophagic marker was stained using anti-p62/CY3. Nuclei were stained with Hoechst. Slides were visualized with Nikon Eclipse Ni-E 60x magnification. Number, size, and intensity of p62 puncta are increased in patient–derived fibroblasts.

**Fig. S4** In silico predictions of the variants p.T105A, p.R335H and p.R370H. The analysis was performed with Dynamut (biosig.lab.uq.edu.au/dynamut/prediction) on the TMEM175 protein structure, PDB accession number 6WCB, chain A. We observed an alteration of the interatomic interactions and of the vibrational entropy energy between wt and mutant proteins. Wt and mutant residues are colored in light green and are represented as sticks alongside with the surrounding residues, which are involved on any type of interactions. Amino acids colored according to the vibrational entropy change upon mutation. Blue represents a rigidification of the structure and Red a gain in flexibility.

**Table S1. The complete list of gene specific primers used for qPCR**

| **Gene** | | **Primer Forward** | | **Primer Reverse** | | **Size** | | **MT** | |
| --- | --- | --- | --- | --- | --- | --- | --- | --- | --- |
| **ATF6** | 5’- AGGCTGGATGAAGATTGGGA-3’ | | 5’-CTGGAGAAAGTGGCTGAGGT-3’ | | 223bp | | 60°C | |  |
| **BIP** | 5’-TTCTTGCCGTTCAAGGTGGT-3’ | | 5’-CTTGGCGTTGGGCATCATTA-3’ | | 208bp | | 60°C | |  |
| **CALR** | 5’-TGATAACTTTGGCGTGCTGG-3’ | | 5’-CTCCTCCTCTTTGCGTTTCT-3’ | | 217bp | | 60°C | |  |
| **CHOP** | 5’-TTAAGTCTAAGGCACTGAGCG-3’ | | 5’-GTCTGATGCCTGTTTTTGTAG-3’ | | 221bp | | 60°C | |  |
| **TMEM175** | 5’-TGCCGTCTACCTGATGACCT-3’ | | 5’-TTGAGCAGGGCAAGTGTGCT-3’ | | 105bp | | 60°C | |  |
| **MTOR** | 5’-CTCGCTTCTATGACCAACTG-3’ | | 5’-TTCCCACCTTCCACTCCTAT-3’ | | 118bp | | 60°C | |  |
| **TFEB** | 5’-CATCAATACCCCCGTCCACT-3’ | | 5’-ACATCATCCAACTCCCTCTC-3’ | | 442bp | | 60°C | |  |
| **SQSTM1** | 5’-GAGATTCGCCGCTTCAGCTT-3’ | | 5’-GGCCATTGTCAATTCCTCGT-3’ | | 204bp | | 60°C | |  |
| **GAPDH** | 5’-TGGTATCGTGGAAGGACTCA-3’ | | 5’-AGGGATGATGTTCTGGAGAG-3’ | | 125bp | | 60°C | |  |

All primer sequences are specific to human genes. MT: melting temperature
