# Supplementary figures and images for: Common and Rare Variants in TMEM175 Gene Concur to the Pathogenesis of Parkinson’s Disease in Italian Patients
Source: Mol Neurobiol. 2023 Jan 7;60(4):2150–73. doi: 10.1007/s12035-022-03203-9 (PMC9984355; doi:10.1007/s12035-022-03203-9)

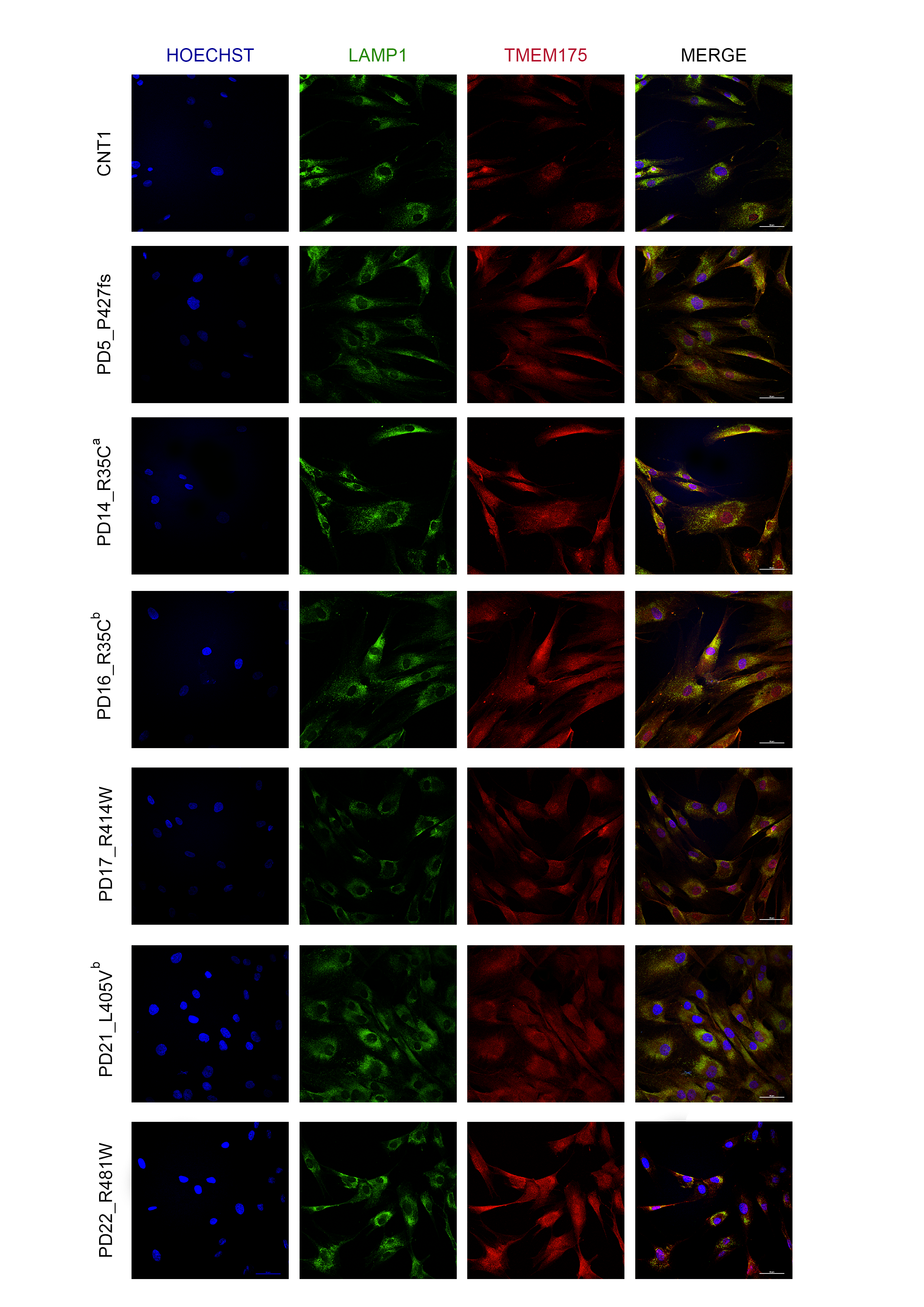

Supplement: Supplementary file 2 — Supplementary file2 (PNG 5354 KB) [file 12035_2022_3203_MOESM2_ESM.png]

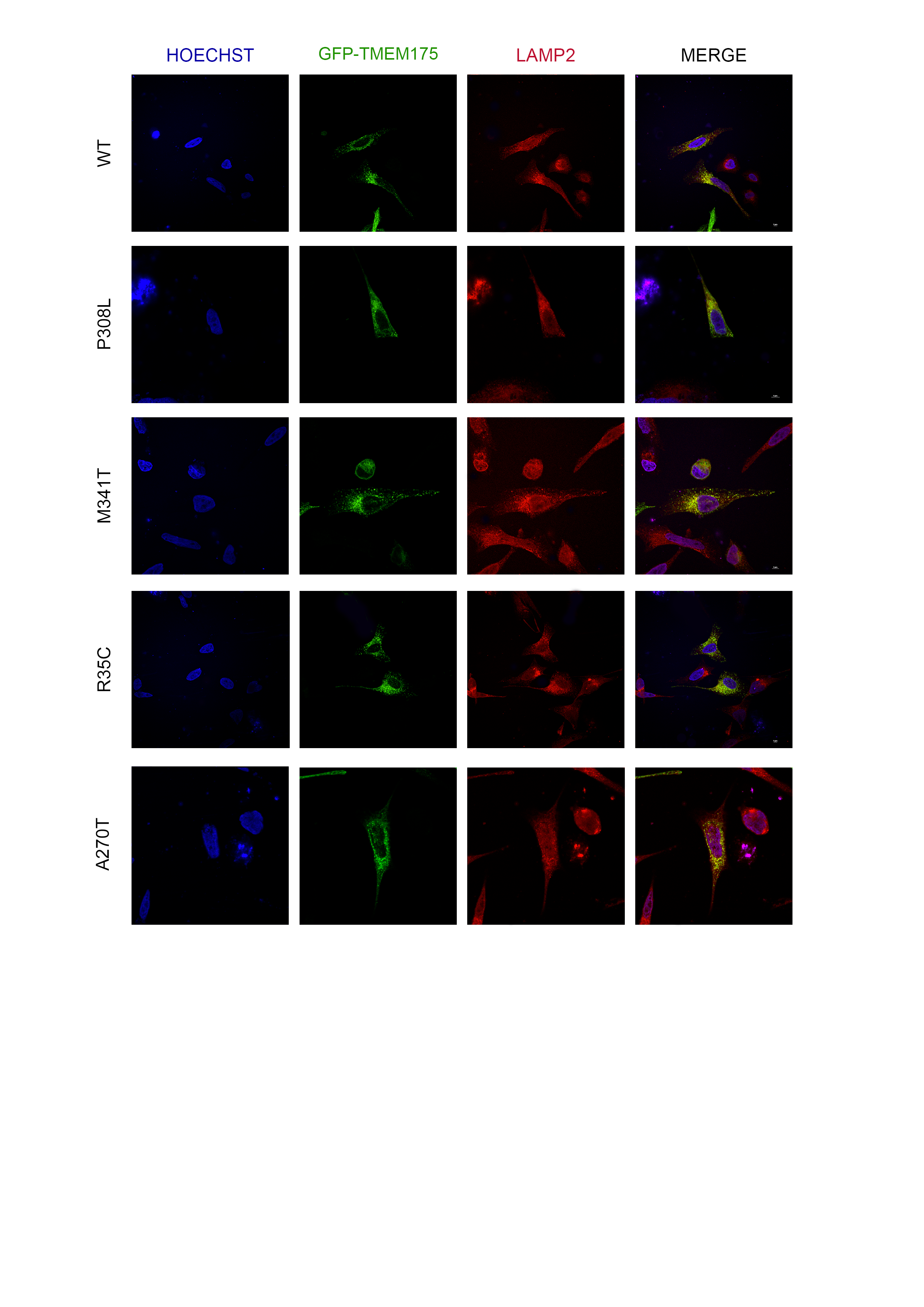

Supplement: Supplementary file 3 — Supplementary file3 (PNG 2468 KB) [file 12035_2022_3203_MOESM3_ESM.png]

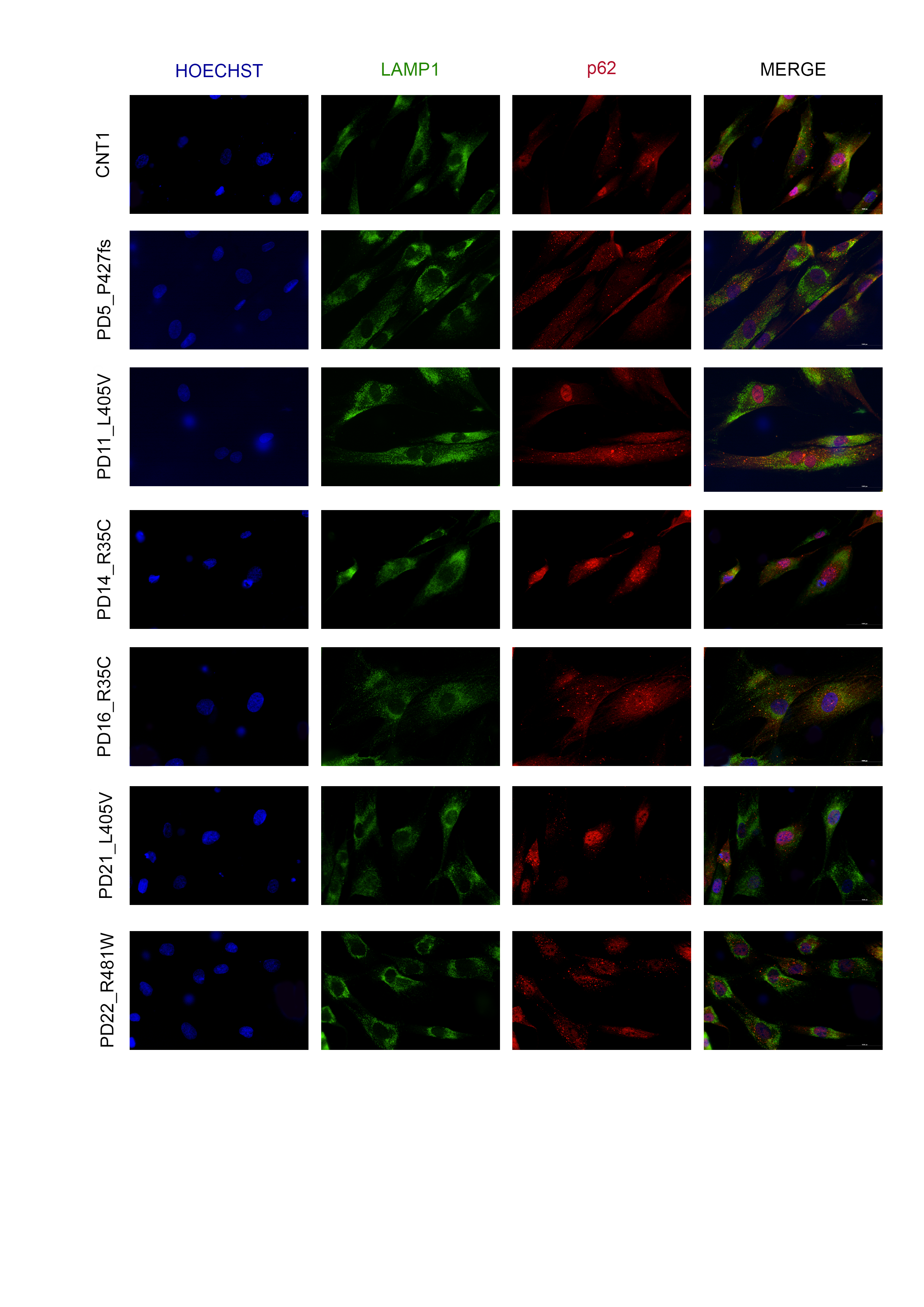

Supplement: Supplementary file 4 — Supplementary file4 (PNG 2544 KB) [file 12035_2022_3203_MOESM4_ESM.png]

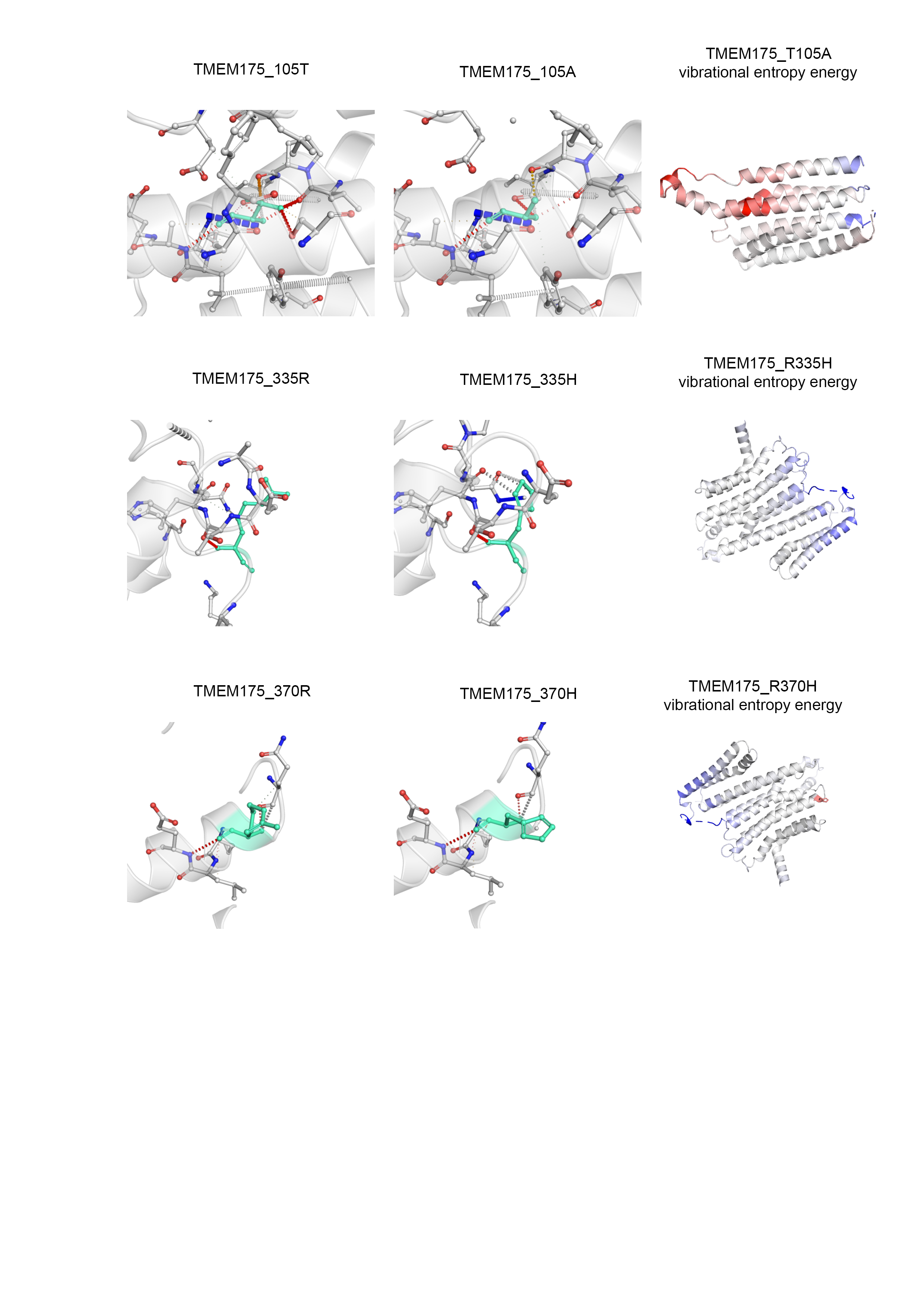

Supplement: Supplementary file 5 — Supplementary file5 (PNG 1249 KB) [file 12035_2022_3203_MOESM5_ESM.png]
